# Proteasome Inhibition Reprograms Chromatin Landscape in Breast Cancer

**DOI:** 10.1158/2767-9764.CRC-23-0476

**Published:** 2024-04-16

**Authors:** H. Karimi Kinyamu, Brian D. Bennett, James M. Ward, Trevor K. Archer

**Affiliations:** 1 Chromatin and Gene Expression Section, National Institute of Environmental Health Sciences, Durham, North Carolina.; 2 Epigenetics and Stem Cell Biology Laboratory, National Institute of Environmental Health Sciences, Durham, North Carolina.; 3 National Institute of Environmental Health Sciences, Durham, North Carolina.; 4 Integrative Bioinformatics Group, National Institute of Environmental Health Sciences, Durham, North Carolina.; 5 Biostatistics and Computational Biology Branch, National Institute of Environmental Health Sciences, Durham, North Carolina.

## Abstract

The 26S proteasome is the major protein degradation machinery in cells. Cancer cells use the proteasome to modulate gene expression networks that promote tumor growth. Proteasome inhibitors have emerged as effective cancer therapeutics, but how they work mechanistically remains unclear. Here, using integrative genomic analysis, we discovered unexpected reprogramming of the chromatin landscape and RNA polymerase II (RNAPII) transcription initiation in breast cancer cells treated with the proteasome inhibitor MG132. The cells acquired dynamic changes in chromatin accessibility at specific genomic loci termed differentially open chromatin regions (DOCR). DOCRs with decreased accessibility were promoter proximal and exhibited unique chromatin architecture associated with divergent RNAPII transcription. Conversely, DOCRs with increased accessibility were primarily distal to transcription start sites and enriched in oncogenic superenhancers predominantly accessible in non-basal breast tumor subtypes. These findings describe the mechanisms by which the proteasome modulates the expression of gene networks intrinsic to breast cancer biology.

**Significance:**

Our study provides a strong basis for understanding the mechanisms by which proteasome inhibitors exert anticancer effects. We find open chromatin regions that change during proteasome inhibition, are typically accessible in non-basal breast cancers.

## Introduction

The 26S proteasome is a large multienzymatic complex, which regulates cellular protein homeostasis by selective degradation of ubiquitinated proteins (1). Proteasome activity is required for many cellular functions and in particular, the proteasome directly regulates chromatin structure and function to influence transcription and gene expression.

Regulation of chromatin function is critical to epigenetic mechanisms that control transcription and gene expression. Chromatin is used by cells to package DNA in the nucleus, a complex process achieved by the compaction of DNA with histone proteins to form nucleosomes, which are the basic units of chromatin (2, 3). The packaging of DNA into chromatin is a key regulatory step for controlling DNA templated processes including transcription (4). Chromatin function is in part dictated by the underlying DNA sequence, and in this regard several processes such as nucleosome remodeling through posttranslational modifications of histones and eviction of nucleosomes to expose underlying regulatory DNA elements can alter the physical properties of chromatin that typically either enhance or repress transcription (4–6).

The proteasome regulates chromatin function at many levels, involving both proteolytic and non-proteolytic activities (7). The expression of many proteins that regulate chromatin dynamics such as chromatin remodeling complexes and histone modifying enzymes are tightly regulated by proteasomal degradation (8). Furthermore, recent evidence suggesting histone proteins are direct targets of proteasomal degradation underscores a critical role for the proteasome in fine tuning chromatin architecture and function (9).

The intimate connection between the proteasome and transcriptional regulation is dynamic and several studies suggest a multifaceted function of the proteasome in transcription regulation that may involve proteolytic and non-proteolytic activities of proteasome complex (10). Non-proteolytic proteasome activities in transcription are supported by studies showing proteasome complex subunits are nuclear proteins and components of the RNA polymerase II (RNAPII) complexes (11–16). The discovery that transcription factor (TF) activation domains often overlap degradation signals lends support for a direct role for proteolytic activities of the proteasome in transcriptional regulation (17). Furthermore, RNAPII itself is a major target of proteasomal degradation, thereby impacting the transcriptional process at every step (18).

Altered chromatin function is linked to aberrant transcriptional regulation in cancer (19, 20). Cancer cells often possess elevated levels of proteasome activity and are therefore more sensitive than normal cells to proteasome inhibitors (21). Proteasome inhibitor drugs have been used for several years to treat several hematologic malignancies, and clinical studies testing their effectiveness in solid tumor cancers are ongoing (22, 23). Their mode of action is generally perceived to be via protein turnover; however, other mechanisms may be at play. A fuller understanding of gene networks that converge to control tumor cell growth and survival in cells exposed to proteasome inhibitors is critical to harnessing their therapeutic potential.

In a recent study, we showed treatment of MCF7 breast cancer cells with proteasome inhibitor MG132 induced and repressed the spreading of H3 lysine methylation and acetylation at promoters of genes that encode tumor suppressors and cell proliferation pathways, respectively (24). Here, we assessed chromatin accessibility, epigenome, and transcriptome dynamics to uncover regions of the breast cancer cell genome that were sensitive to proteasome inhibition. We uncovered profound changes in chromatin accessibility, the epigenome, and transcriptome dynamics in breast cancer cells in response to proteasome inhibition. We found that proteasome activity is required for the accessibility of specific *cis*-regulatory elements in the MCF-7 breast cancer cell genome. Changes in accessible chromatin in treated cells were associated with enriched active histone marks and differences in RNAPII transcription. Chromatin changes distal to gene promoters upon MG132 treatment underscore the proteasome's role in regulating chromatin state and RNAPII transcription from *cis*-regulatory elements critical for tumor maintenance.

## Materials and Methods

### Experimental Model System and Treatment Conditions

#### Cell Line and Cell Culture

MCF-7 (HTB-22) breast cancer cells authenticated by short tandem repeat profiling were directly purchased from ATCC (MCF7 – HTB-22 | ATCC). Cells were maintained in a humidified incubator at 37°C with 5% CO_2_ in Modified Eagle Medium (MEM, GIBCO) supplemented with 10% FBS (Atlanta Biologicals), 100 µg/mL penicillin/streptomycin, 2 mmol/L glutamine, and 10 mmol/L HEPES (GIBCO). MCF-7 cells were routinely tested for *Mycoplasma* contamination by the Quality Assurance Laboratory (NIEHS, RTP, NC), testing negative most recently, February 2024. Briefly, an aliquot of cells was aerobically cultured on 5% sheep blood agar for 24 hours at 37°C. *Mycoplasma* contamination was assayed using the MycoAlert Mycoplasma Detection Kit (catalog no. LT07-318, Lonza), followed by detection using real time PCR (IDEXX BioResources).

#### Experimental Conditions for Inhibiting Proteasome Activity

MG132 is a small molecule that effectively blocks the proteolytic activity of the 26S proteasome complex. To inhibit proteasome activity, MCF-7 breast cancer cells were seeded for 24 hours in phenol red-free MEM (GIBCO) supplemented with 5% charcoal-stripped calf serum (Atlanta Biologicals), 10 mmol/L HEPES, and 2 mmol/L glutamate. After 24 hours, cells were switched to medium containing vehicle (DMSO, Sigma) as control or 1 µmol/L MG132 (Calbiochem) for 4 and 24 hours. For all experiments, MCF-7 cell culture did not exceed passage 25.

### Transcription and Gene Expression Analysis

#### RNA Extraction

Total RNA was extracted from three biological replicates of vehicle and MG132-treated cells using total RNA isolation kit (Norgen Biotek). RNA concentration was determined using a Nanodrop ND-1000 spectrophotometer (Thermo Fisher Scientific). The integrity of the RNA sample was verified using the Agilent RNA 6000 kit and Agilent Bioanalyzer 2100 (Agilent Technologies). Samples with an RNA integrity number equal or above 8 were considered appropriate for further downstream analysis.

#### mRNA Expression Quantification by RNA sequencing

RNA sequencing (RNA-seq) of ribosomal-depleted RNA was performed by Expression Analysis, Q^2^ Solutions (Morrisville, NC). Libraries were sequenced to a depth of an average of 60 million reads (paired end, 2 × 75 bp). Raw reads were quality filtered to include only those with a mean Phred quality score of 20 or greater. Adapter was trimmed using Cutadapt version 1.12. The preprocessed reads were aligned to the hg19 assembly using STAR version 2.6.0 (25). The STAR index was built using a GTF file derived from GENOCDE v27 and –sjdbOverhang 10. Read counts were generated using the featureCounts tool from the Subread package version 1.5.1. Differentially expressed genes (log_2_ fold change of ±1; adjusted *P*-value ≤ 0.05) were detected with the DESeq2 package (26).

#### Analysis of RNAPII Genome Occupancy to Quantify Transcriptional Activity

RNAPII genome occupancy was used as a surrogate to measure transcription rates across the genome. Chromatin immunoprecipitation and sequencing (ChIP-seq) assays were performed to identify regions of the genome enriched with RNAPII in MCF-7 cells treated with MG132. Cells were treated with vehicle or MG132 as specified above, and chromatin immunoprecipitation (ChIP) for RNAPII done as follows: MCF-7 cells were briefly cross-linked in 1% formaldehyde PBS for 5 minutes. After 5 minutes, the cross-link was quenched with glycine (125 mmol/L) for 5 minutes. Cells were pelleted by centrifugation and resuspended in sonication buffer (20 mmol/L Tris 8.0, 150 mmol/L NaCl, 0.5% Triton X-100, 2 mmol/L Ethylenediaminetetraacetic acid (EDTA), 10% glycerol) supplemented with protease inhibitors and incubated on ice for 10 minutes. Chromatin was fragmented in 15 mL tubes using the bioruptor (Diagenode) for 12 cycles (30 seconds on/30 seconds off), total sonication time 6 minutes. After sonication, the fragmented chromatin was spun briefly in a cooled centrifuge, transferred to 1.5 mL Eppendorf tubes, and centrifuged at 14,000 RPM for 10 minutes. Following centrifugation, an aliquot of the chromatin was diluted with immunoprecipitation buffer and incubated with various RNAPII antibodies for 12–14 hours at 4°C. DNA/protein immunocomplexes were recovered as described below for histone modification ChIP.

### Analysis of Annotated and Unannotated Transcription from *Cis*-regulatory Elements

#### Start Sequencing Analysis to Detect Short Capped RNAs

Start sequencing (start-seq) quantifies short, capped, chromatin-associated RNAs, which are indicative of newly synthesized nascent RNA. It is a useful method for detecting transcription from *cis*-regulatory elements, including promoter and enhancer elements. Short capped RNAs were prepared as described previously (27) with some modifications (28). Approximately 20 million nuclei were isolated from control and MG132-treated cells using hypotonic lysis buffer followed by total RNA extraction using TRIzol reagent (Invitrogen). Small RNAs, 25–80 nt, were size selected on 15% Urea-TBE gel (Novex). Gel slices were crushed by centrifugation (16,000 × *g*) and RNA eluted following incubation in 300 mmol/L NaCl at room temperature for 2.5 hours. The eluted sized RNA was separated from the gel slices by short centrifugation using cellulose acetate spin filters (Agilent, catalog no. 5185-5990). The 5′-triphosphates on these RNAs were converted to monophosphates by treating the short nuclear RNA 5′ Polyphosphatase. The digestion reaction was purified using Zymo Oligo clean and concentrator kit. The eluted RNA was then treated with 5′ Terminator exonuclease enzyme (Epicentre) to remove the uncapped RNA, and capped RNAs were recovered using the ZYMO kit as above. Sequencing libraries were prepared using Illumina TruSeq small RNA as per the manufacturer's instructions, with the ligation of 3′- small RNA Tru-Seq adapter using the truncated T4 RNA Ligase 2 (NEB). A total of 45–150 nt capped small RNAs were recovered on 15% Urea-TBE gel (Novex) and RNA was extracted from the gel as described above. The RNA 5′ ends were dephosphorylated using Heat Labile Alkaline phosphatase (Epicentre), purified using ZYMO columns and treated with RNA 5´ Pyrophosphohydrolase (RppH) to create 5´ monophosphate RNA, followed by another column clean up and elution of 5′ ends dephosphorylated RNA. For small RNA-seq, 5′-Tru-Seq small RNA adapters were ligated on to the decapped RNA with T4 RNA Ligase 1 (NEB) in the presence of ATP and cDNA synthesized following Illumina's small RNA-seq protocol. Small RNAs were enriched by PCR amplification and size separated on 6% native gel (Novex). Gel slices corresponding to RNA sizes between 100 and 200 bp were excised, and RNA purified using Qiagen MinElute kit. Concentration of the library was determined using Qubit and RNA sequenced using a NextSeq 500 system (Illumina).

### Analysis of Chromatin State

#### Chromatin Accessibility

##### Assay for Transposase-accessible Chromatin and Sequencing

Assay for Transposase-accessible Chromatin and sequencing (ATAC-seq) uses a hyperactive Tn5 transposase to insert Illumina sequencing adaptors into accessible chromatin regions in the genome. ATAC-seq is a powerful approach to identify potential regulatory elements in the whole genome. To perform ATAC-seq, MCF-7 nuclei were prepared as originally described with minor modifications (29). Briefly, 50,000 cells were pelleted and resuspended in ice-cold cytoskeletal lysis buffer, 10 mmol/L PIPES pH 6.8, 100 mmol/L NaCl, 300 mmol/L sucrose, 3 mmol/L MgCl_2_, 0.1% TritonX-100 (30). Cells were incubated on ice for 5 minutes and spun at 500 × *g* for 5 minutes at 4°C, followed by digestion with 5 µL of transposase (Nextra DNA kit; refs. 29, 30). Transposase digestion was performed for 30 minutes at 37 °C, on a block shaker set at 200 rpm. Digested DNA was purified using MinElute PCR purification kit (Qiagen) and eluted from the MinElute columns with a 10 µL volume of Elution Buffer (Qiagen). Accessible DNA libraries were amplified by PCR using NEBNext High-Fidelity PCR Master Mix (New England Biolabs), with custom Nextera PCR primers as described previously (29). Libraries were amplified for a total of 10 PCR cycles after verifying in each case the number of cycles for optimal amplification. DNA purification and size selection were performed using AmpureXP SPRI select beads (Beckman Coulter). Fragmented DNA was eluted in 30 µL elution buffer (Qiagen PCR kit). An aliquot of the DNA was resolved on 1% agrose gel and visualized on ChemiDoc Touch Imaging System (Bio-Rad) to determine the quality of the fragmented libraries. Samples were subjected to paired-end sequencing using 2  ×  50 bp reads on an Illumina NextSeq High2000 at the NIEHS Epigenomics core. Three independent biological replicates were performed for each treatment condition, generating a total of nine samples.

### Analysis of Epigenetic Modifications

#### Detection of Histone Modification Patterns

ChIP-seq assays were performed to identify regions of the genome enriched with specific histone modifications in MCF-7 cells treated with MG132. Cells were treated with vehicle or MG132 as specified above. Chromatin immunoprecipitation for histone modifications was done as follows: MCF-7 cells were briefly cross-linked in 1% formaldehyde PBS for 5 minutes. After 5 minutes, the cross-link was quenched with glycine (125 mmol/L) for 5 minutes. The quenched cross-linker was quickly discarded, and cells were rinsed twice with PBS supplemented with protease inhibitors. Cells were scraped and harvested in 5 mL PBS with an additional 5 mL PBS rinse to collect most of the cells. The cells were pelleted by centrifugation at 4°C, followed by nuclei isolation as described previously (31). To fragment chromatin, nuclei were resuspended in micrococcal nuclease (MNase, Worthington Enzymes) digestion buffer (10 mmol/L Tris-HCl pH 7.4, 15 mmol/L NaCl, 60 mmol/L KCl, 1 mmol/L CaCl_2_, 0.15 mmol/L spermidine, 0.5 mmol/L spermidine) on ice. Nuclei were digested with 50 units of MNase nuclease on a temperature-controlled heating block at 25°C for 20 minutes with gentle mixing at 400 rpm. Following digestion, samples were quickly placed on ice and the reaction stopped by addition of EDTA/Ethyleneglycol-bis(β-aminoethyl)-N,N,N′,N′-tetraacetic acid (EGTA) stop buffer (100 mmol/L, EDTA, 10 mmol/L EGTA, pH 7.5), gently mixed by pipetting, followed by addition of SDS lysis buffer (4%SDS, 40 mmol/L EDTA, 200 mmol/L Tris pH 8.0), final concentration 1% SDS supplemented with Halt Protease Inhibitor Cocktail (Thermo Fisher Scientific). To further release chromatin, nuclei were briefly disrupted using a mini homogenizer for 5 seconds and further incubated on the Hulamixer sample mixer (Invitrogen) for 10 minutes at 4°C. Chromatin was recovered by centrifugation at 14,000 rpm for 10 minutes. Following centrifugation, an aliquot of MNase fragmented chromatin was diluted 10X with immuno precipitation buffer (20 mmol/L Tris 8.0, 150 mmol/L NaCl, 0.5% Triton X-100, 2 mmol/L EDTA, 10% glycerol) supplemented with protease inhibitors (Roche). Antibodies against histone modifications of interest were added and chromatin incubated overnight at 4°C on a slow rotating nutator. The next day, 20 µL Protein A and G magnetic Dynabeads (Invitrogen) were added, and samples were incubated for an additional 2 hours at 4°C on nutator to capture DNA/protein immunocomplexes. Following incubation, protein/DNA immunocomplexes were recovered by subsequent washes and eluted as described previously (32). Eluted immunoprecipitated complexes were digested with RNAse A (Qiagen) followed by proteinase K digestion and reverse cross-linking as described previously (32). DNA was recovered using Qiagen PCR kit purification system (Qiagen) and quantified using Quant-iT dsDNA HS assay kit with Qubit fluorometer (Invitrogen).

### ChIP-seq Library Preparation

Following DNA recovery from RNAPII and histone modification immunocomplexes, libraries were prepared with Illumina compatible NEXTflex Rapid DNA-seq Kit and sequenced on a NextSeq2000 (Illumina). At least two independent biological replicates were performed for each histone modification and RNAPII chromatin immunoprecipitation assays. Reagents and antibodies used for this study are reported on key resource Supplementary Table S1.

### ChIP-seq Processing

The FASTQ files for each biological replicate were concatenated for each sample. Raw reads were quality filtered to include only those with a mean Phred quality score of 20 or greater. Adapter was trimmed using Cutadapt version 1.12. The preprocessed reads were aligned to the hg19 assembly using Bowtie version 1.2 and parameters -v 2 -m 1 –best –strata (33). Aligned reads were deduplicated by only keeping one read pair when multiple pairs had both mates aligned to the same position. Bound locations were obtained from the aligned reads by extracting the entire length of the aligned fragment. Coverage tracks were generated from these bound locations using the genomecov tool from the bedtools suite version 2.17.0 (34). The coverage tracks were normalized to depth per 10 million mapped reads.

### Analysis of Superenhancers

Additional peaks were called for the H3K27ac ChIP-seq samples using MACS2 and parameters -q 0.000001 –fe-cutoff 5. Superenhancers (SE) were identified from these peaks using ROSE and parameter -t 2500 (35).

### ATAC-seq Data Processing

Raw reads were quality filtered to only include those with a mean Phred quality score of 20 or greater. Adapter was trimmed using Cutadapt version 1.12. The preprocessed reads were aligned to the hg19 assembly using Bowtie version 1.2 and parameters -v 2 -m 1 –best –strata. Reads aligning to the mitochondrial chromosome were removed. Surviving reads were deduplicated by only keeping one read pair when multiple pairs had both mates aligned to the same position. Nucleosome-free reads were extracted by requiring the fragment length to be less than 100 bases. All heat maps, metaplots, and downstream analyses were done using the coverage from these nucleosome-free reads. Coverage tracks were generated using the genomecov tool from the bedtools suite version 2.17.0. The coverage tracks were normalized to depth per 10 million mapped reads. Peaks were called independently for each sample using MACS2 v2.1.0 and parameters -q 0.0001 –nomodel –extsize 9. Peaks for all samples were merged, and any resulting merged peaks that were within 100 bases of each other were also merged, resulting in a set of global open chromatin regions (OCR). Counts for each sample and OCR were obtained by counting the number of reads that overlap with the OCRs. Differential open chromatin regions (DOCR) were identified using DESeq2, requiring an FDR of less than 0.05 and at least 100 reads in each sample for that OCR. A DOCR was categorized as promoter if its center fell with the region 1 kb upstream to 500 bases downstream of an annotated transcription start site (TSS). Remaining DOCRs were categorized as genic if the center fell within the gene body of an annotated TSS, and all other DOCRs were categorized as intergenic. Epilogos chromatin state scores in 200-base bins were used to define consensus chromatin states with highest score in each bin (36). DOCRs were annotated using the 15 Epilogos chromatin states, based upon overlap with the center of each DOCR. DOCRs were summarized by direction of change, genome category, and chromatin state.

### Heat map and Metaplot Processing

Signal used in heat maps and metaplots (for all data types) was derived from the coverage of aligned fragments. All signal (except for start RNA) was normalized to “depth per 10 million mapped fragments,” by multiplying the coverage signal by 10 million and dividing by the number of aligned fragments. Heat maps were generated by specifying 100 bins covering the genomic regions in the heat map, and by calculating the average normalized signal in those genomic regions for each bin. Histone modification ChIP-seq heat maps were further normalized by first generating a similar heat map with total H3 signal, then subtracting the total H3 signal from the histone modification ChIP-seq signal for each bin. RNAPII ChIP-seq heatmaps were normalized in a similar way, except that input signal was subtracted. All difference heat maps were generated by taking the two original heat maps and subtracting the signal in each bin. Metaplots were created by averaging the signal across all features for each bin in the corresponding heat map.

### Motif Analysis

Motif enrichment analysis was performed using CentriMo v4.12.0 from the MEME suite. Enrichment was searched for within the regions 1 kb upstream to 1 kb downstream of the centers of the DOCRs. Motifs tested were the “HOCOMOCOv11_core_HUMAN” set obtained from the MEME motif database v12.21. Footprinting analysis was performed using the TOBIAS suite v0.13.1 with the ATACorrect, FootprintScores, and BINDetect tools. Motifs used with TOBIAS were the “JASPAR2022_CORE_vertebrates_non-redundant_pfms” set obtained from the JASPAR database.

### Pathway Analysis

DOCR regions were associated with the nearest upstream or downstream gene, subdivided by DOCR direction (GAIN, LOST) and genomic context (genic, non-genic), generating four sets of genes: GAIN genic, GAIN non-genic, LOST genic, LOST non-genic. Each gene set was tested for hypergeometric enrichment using clusterProfiler-4.6.2 (37) with gene sets defined by MSigDB obtained using msigdbr-7.5.1 (https://CRAN.R-project.org/package=msigdbr). Enrichments were tested separately using Gene Ontology Biological Process (gobp), Gene Transcription Regulation Database (GTRD), and Hallmark Pathways. Pathways with adjusted *P*-value 0.05 and at least four genes were used for multienrichment analysis with R Github “jmw86069/multienrichjam” (38). The four sets of pathways were integrated to form a pathway-gene incidence matrix, from which four functional clusters were defined using hierarchical clustering. Pathway clusters and genes were represented as a concept network, color-coded by input gene set. Genes with significant expression changes by RNA-seq were indicated with a colored border around each gene node.

### Biological Relevance of MG132 DOCRs

Processed ATAC-seq coverage files were downloaded for 74 The Cancer Genome Atlas (TCGA) breast invasive carcinoma (BRCA) tumor samples (39). Signal from technical replicates was merged for each sample using unionBedGraphs from the BEDTools suite.

### TCGA Breast Cancer ATAC Signal Heat Maps

Signal coverages were summarized for each TCGA sample, at DOCR positions which overlapped the called SEs. Samples were split into basal or non-basal subgroups (39). Data were visualized using ComplexHeatmap (40) with sample columns, and SE DOCRs rows, displaying row-centered signal. Samples were split into basal or non-basal subgroups, and rows were split into six subclusters after hierarchical clustering.

### Estrogen Receptor ChIP-seq Signal Heat Maps

Estrogen receptor (ER) ChIP-seq signals from ref. 41 were summarized at DOCRs overlapping called SEs, by quantifying reads across 20 bins scaled evenly across each SE region, with 2,000 bases upstream and 2,000 bases downstream the SE region each divided into 10 200 base bins. ER ChIP-seq signals were adjusted by subtracting input signal from ER-IP signal within each treatment group, estradiol (E2) or vehicle. Adjusted signals were displayed as heat maps, with the same row ordering and subclusters defined by TCGA breast cancer heat maps.

### Start-seq Data Processing

Raw reads were quality filtered to include only those with a mean Phred quality score of 20 or greater. Adapter was trimmed using Cutadapt version 1.12 (42). The preprocessed reads were aligned to the hg19 assembly using Bowtie version 1.2 and parameters -v 2 -m 1 –best –strata (33). TSS calls were assigned from the aligned reads using GENCODE v27 and the TSScall tool. TSScall is based on methods used in previous studies (43), and it is available on GitHub (https://github.com/lavenderca/TSScall). In addition to identifying TSS locations, this tool also categorized the TSS calls as either associated with an annotated TSS (obsTSS) or not associated with an annotated TSS (uTSS), and it identified whether the TSS calls have a downstream antisense (divergent) TSS. This information was used to further categorize the obsTSSs as either having a divergent TSS that was also another obsTSS (bidirectional) or having a divergent TSS that was a uTSS (PROMPT). Also, any uTSS that overlapped any part of a gene body was further categorized as genic, whereas all other uTSSs were categorized as intergenic.

### Data Availability

The datasets supporting the results and conclusions of this article are available in the Gene Expression Omnibus repository, GSE241601, GSE241597, GSE241598, GSE241599, GSE241600. All other data in this article can be obtained from the corresponding author upon reasonable request.

## Results

### Proteasome Inhibition Reprograms Accessible Chromatin

**FIGURE 1:**
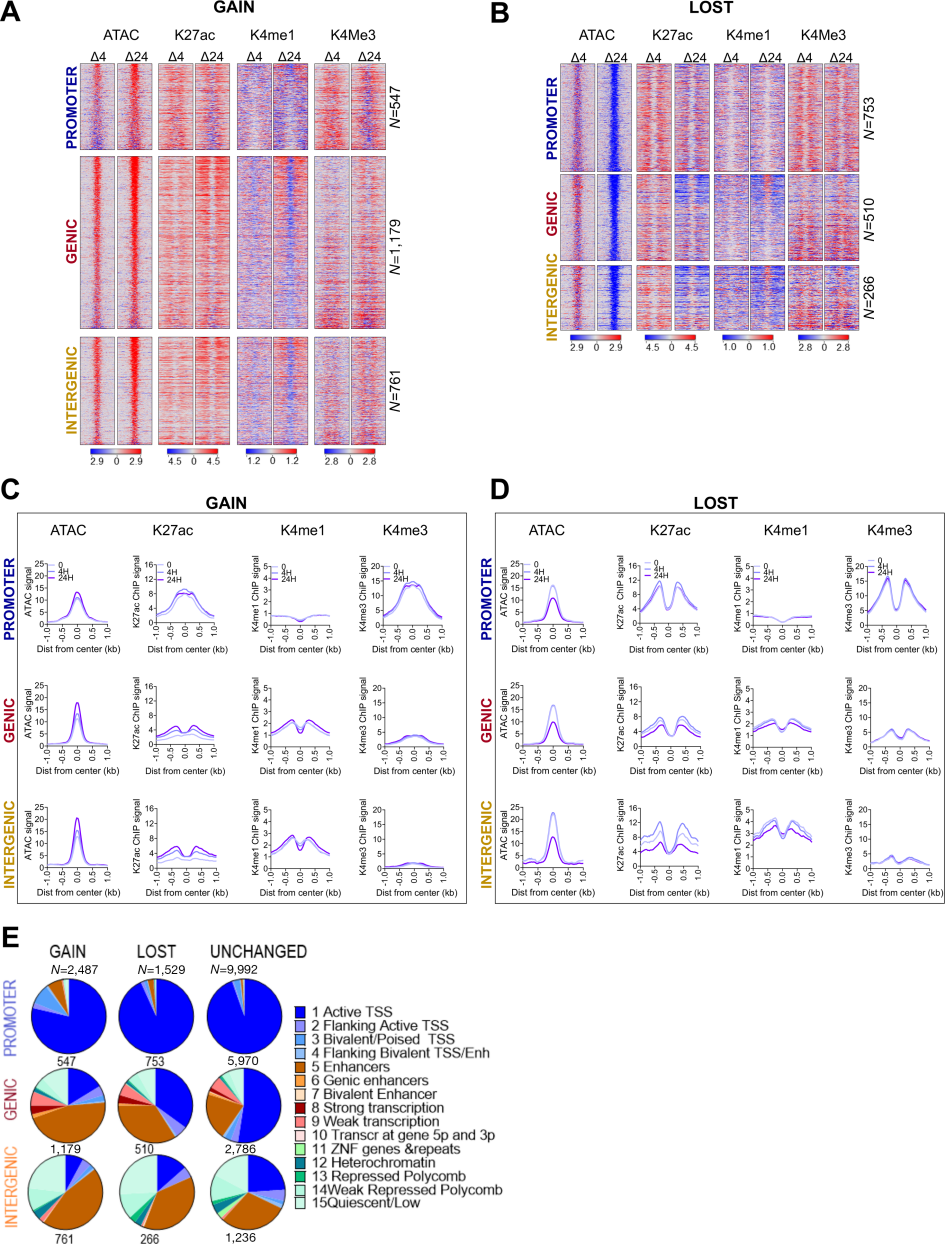
Proteasome inhibition reprograms accessible chromatin. **A,** Heat maps showing differential (MG treated minus untreated) signal for DNA accessibility (ATAC, nucleosome-free reads <100 bp), H3K27ac (K27ac), H3K4me1 (Kme1), and H3K4me3 (K4me3) at DOCRs that increase **(GAIN)** accessibility after 24H treatment compared with untreated. **B,** Heat maps as in A at DOCRs that decrease **(LOST)** accessibility. Signal spans ±1 kb from the center of the defined DOCR regions and is ranked on the basis of the degree of change in accessibility, where regions at the top have the most change in accessibility. The color scale shows an increase (red) or decrease (blue) in differential signal. Side by side heat maps show differential signal at the 4H and 24H time points. DOCRs are split by genomic category into **PROMOTER**, **GENIC**, and **INTERGENIC**. *N* is the number of DOCRs in each genomic category. **C,** Metaplots for ATAC signal and ChIP-seq binding signals of K27ac, K4me1, and K4me3 at **GAIN** DOCRs. **D,** Metaplots as in C, **LOST** DOCRs. Signal includes the untreated state (0) and after 4H and 24H of treatment. Line color density reflects treatment conditions [light (0) to darkest 24H)]. **E,** Pie charts showing the proportion of DOCRs assigned to various chromatin states using Epilogos https://epilogos.altius.org/. *N* is the number of DOCRs in each category.

Chromatin accessibility was assessed by Assay for Transposase-Accessible Chromatin with high-throughput sequencing (ATAC-seq; ref. 29) after 0-, 4-, and 24-hour treatment with MG132 resulting in a total of 162,546 merged peaks from which a subset of 14,008 high-confidence OCRs were tested for differential accessibility (Fig. 1; Supplementary Fig. S1A--S1O; Supplementary Table S2).

Of the 14,008 OCRs tested, 346 and 4,016 were increased or decreased, DOCRs in cells treated with MG132 for 4 and 24 hours, respectively (Supplementary Fig. S1A–S1D; Supplementary Table S2). The size distribution of OCRs was similar for unchanged and differential regions, suggesting that the size of the regions was not the key factor determining the differences in DNA accessibility (Supplementary Fig. S1D). The distribution of distances to nearby TSSs of the ATAC peaks did not differ between treatment conditions (Supplementary Fig. S1E). However, while 65% of the aggregate tested OCRs were close to a TSS (<±5 kb), only 10% (4H) and 40% (24H) of the DOCRs were close to a TSS (Supplementary Fig. S1F). To understand how the differences in accessibility are associated in a genomic context, we classified the DOCRs based on their genomic space: proximal to a TSS (promoter), within the transcriptional unit of a gene (genic), or outside of a gene (intergenic; Supplementary Fig. S1G). We found regions with increased accessibility were predominantly in the genic and intergenic space, while a higher percentage of those with decreased accessibility were promoter proximal (−1 to +0.5 kb; Supplementary Fig. S1G). In addition, although there was a slight bias toward DOCRs with increased accessibility (GAIN) across chromosomes, three chromosomes (Chr 16, 19, and 22) were primarily enriched for DOCRs with decreased accessibility (LOST; Supplementary Fig. S1H).

There was a time-dependent change in accessibility following MG132 treatment, with most of the changes occurring in cells treated with MG132 for 24 hours. Also, regions that showed an increase in accessibility during the 4-hour treatment largely overlapped with those that increased accessibility during the 24-hour treatment (Supplementary Fig. S1I). For this reason, downstream analysis was performed with the 4,016 regions following 24-hour treatment, of which 2,487 were more accessible (GAIN) and 1,529 were less accessible (LOST; Supplementary Table S2; Supplementary Fig. S1J). Regions that became more accessible showed a progressive increase in DNA accessibility from 4H to 24H (Supplementary Fig. S1J). In contrast, the less accessible regions, show little decrease at 4H and do not appear to significantly decrease until 24H (Supplementary Fig. S1J). This result suggests that genomic regions that become accessible following MG132 start to do so rapidly (within 4H), whereas regions that become less accessible start to compact later.

Further analysis revealed distinct differences in accessibility when comparing GAIN and LOST DOCRs. GAIN DOCRs showed a larger magnitude of change in the genic and intergenic space compared with the promoter proximal regions [(Fig. 1A and C), compare ATAC enrichment shown on metaplot, 24H vs. 0 lines]. In contrast, in the LOST DOCRs, all three genomic regions show a similar decrease in DNA accessibility (Fig. 1B and D). In addition, at 24 hours, promoter proximal regions that increase accessibility do not reach the same level of openness as of those that decrease accessibility in untreated cells [compare the scale ATAC GAIN 24H (Fig. 1C) with ATAC LOST 0H (Fig. 1D)]. Thus, following MG132 treatment, genomic context influences the magnitude of change in chromatin accessibility and decompaction of chromatin occurs sooner than compaction, ultimately leading to reprogramming of accessible sites in the genome.

### *Cis*-regulatory Elements Acquire Distinct Chromatin Features for Gained and Lost Accessibility

DNA accessibility is regulated through the actions of multiple histone posttranslational modifications (5). Some well-characterized posttranslational modifications of histones colocalize with specific chromatin states. Because a majority of DOCRs were distal to TSSs, we interrogated *cis*-regulatory control elements using ChIP-seq with antibodies against H3K4me3, H3K27ac, and H3K4me1 histone marks, generally enriched at promoter and enhancer elements, respectively (refs. 44, 45; Fig. 1A–D).

The H3K27ac mark was differentially associated with both GAIN and LOST DOCRs. Promoters were generally enriched with H3K27ac and H3K4me3, but largely depleted of H3K4me1 (Fig. 1). Furthermore, promoter regions with increased accessibility showed a distinct chromatin architecture compared with those with decreased accessibility, based on differences in the shape of the H3K27ac and H3K4me3 signals (Fig. 1C and D). In regions that increase accessibility, H3K27ac and H3K4me3 accumulate at the center of the accessible region, exhibiting a unimodal profile. However, in regions that decrease accessibility, both marks flank the center of the region, in a bimodal profile, which is generally associated with accessible regions and particularly canonical promoters (Fig. 1D; Supplementary Fig. S1K–S1M). Analysis of the enrichment plots derived from mononucleosome fragments, total H3, H3.3, and nucleosome-free fragments, reveal additional differences between the two classes of promoters (Supplementary Fig. S1N). At promoters with increased accessibility, the mononucleosome signal is enriched at the center of the region but depleted in regions with decreased accessibility. Compared with LOST, GAIN promoters show lower accessibility (ATAC), higher total H3 and lower H3.3 signal though enriched with H3K122ac at center of the region. Taken together, these results demonstrate distinctive effects on chromatin architecture at promoters that increase compared with those that decrease DNA accessibility following MG132 treatment.

DOCRs at genic and intergenic regions were also differentially enriched with histone marks. Overall, the largest changes in H3K27ac were observed in genic and intergenic regions, where H3K27ac was enriched and depleted at regions that increase and decrease accessibility, respectively, consistent with the direction of chromatin accessibility (Fig. 1). Similarly, genic and intergenic regions were also enriched or depleted of H3K4me1, indicating gain and loss of enhancer activity, respectively. In addition, H3K27ac-enriched genic and intergenic regions predominantly displayed bimodal profiles, irrespective of DOCR class. In contrast, H3K4me1 was depleted and enriched at the center of GAIN and LOST DOCR, respectively, indicating differential states of putative enhancers (Fig. 1C and D). Genome browser shots represent examples of genomic loci showing distinct differences in the chromatin landscape of DOCR classes (Supplementary Fig. S1O).

We also explored the relationship of DOCR directionality with genomic region and Epilogos chromatin state data (http://epilogos.altius.org/; ref. 36; Fig. 1E). Promoter regions were consistently associated with the transcriptional regulatory function of active TSS (state 1), however there were differences in chromatin architecture between GAIN and LOST promoters. GAIN promoters showed a higher enrichment of states marked by bivalent/poised TSS and enhancer states (state 3 and 5), whereas LOST promoters look more like unchanged promoters, representative of canonical promoters (Fig. 1E).

Further analysis of chromatin states also revealed additional differences in chromatin architecture of genomic regions affected by MG132. Genic regions with increased accessibility were associated with fewer transcriptionally active states (state 1–4) compared with regions with decreased accessibility, but both DOCR classes overlapped proportionally more enhancer regions than unchanged regions (state 5–7; Fig. 1E). DOCRs in intergenic space were associated with enhancer states (states 5–7) more so than unchanged regions, while all three classes of intergenic OCRs showed association with repressive chromatin states (states 11–14; Fig. 1E). In summary, integrating ATAC-seq and ChIP-seq of histone marks revealed distinct chromatin architecture of promoter DOCRs and uncovered a subset of genic and intergenic regions associated with putative active and poised enhancers in MG132-treated cells.

### Differentially Accessible Chromatin Affects Transcription Initiation

**FIGURE 2:**
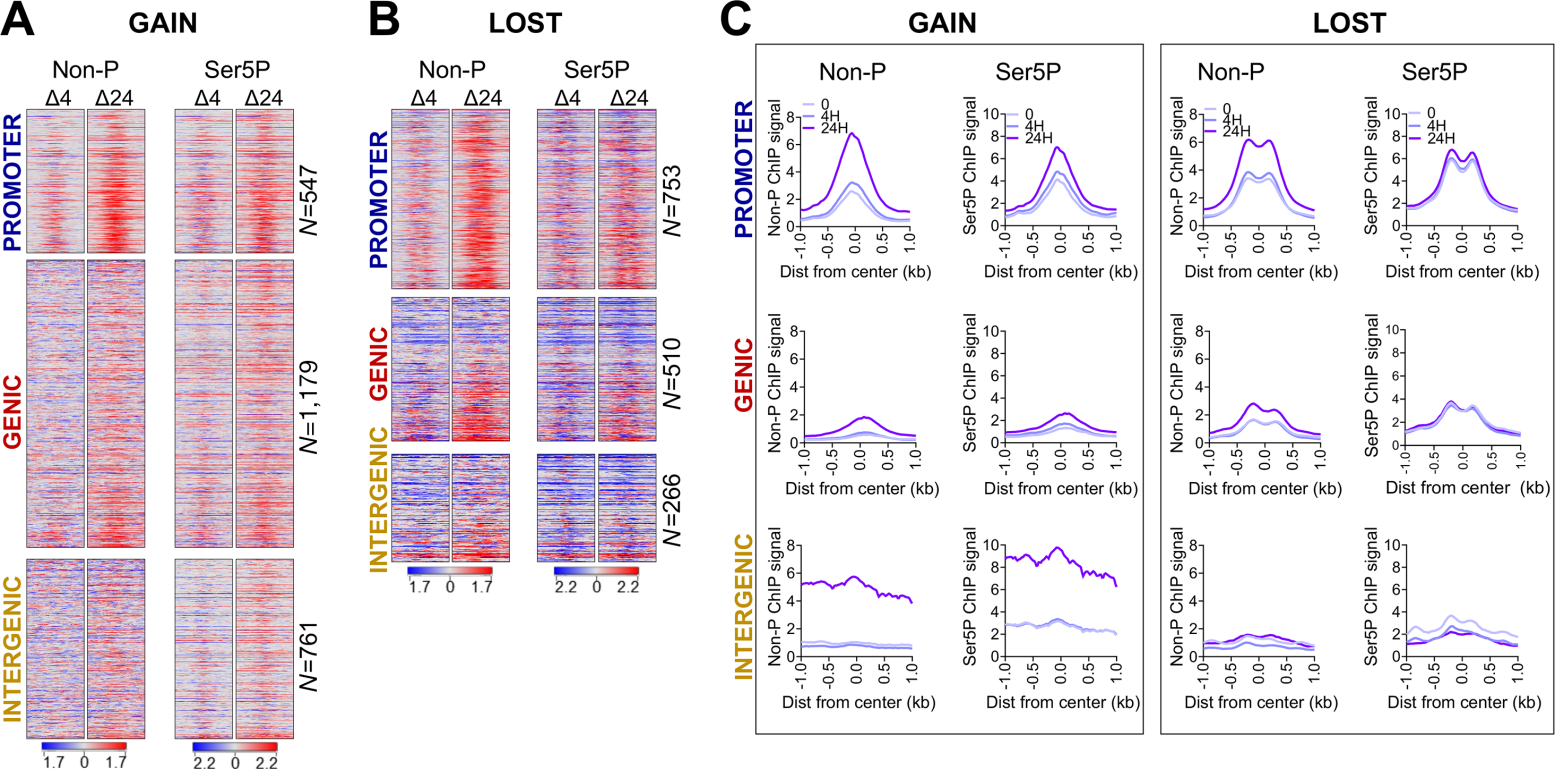
Proteasome inhibition affects RNAPII binding. Heat maps showing differential signal for non-phosphorylated (Non-P), and serine-5 phosphorylated (Ser5P) RNAPII at DOCRs that increase **(GAIN; A)** and decrease (**LOST; B**) accessibility. Signal spans ±1 kb from the center of the DOCRs and is ranked on the basis of the degree of change in accessibility. **C,** Metaplots for ChIP-seq binding signals of Non-P and Ser5P RNAPII at DOCRs. Signal includes the untreated state (0) and after 4H and 24H of treatment.

Chromatin impedes transcription and the dynamic changes in chromatin accessibility that we observe in MG132-treated cells may have potential consequences on transcription. We next performed ChIP-seq with antibodies against RNAPII to profile the genome-wide occupancy of RNAPII as a proxy for transcription in MG132-treated cells (Fig. 2). Regions that increase accessibility upon treatment were enriched with RNAPII, where the largest changes in Pol II binding occurred at promoter and to a lesser degree at intergenic DOCRs (Fig. 2A). Regions with decreased accessibility exhibited different RNAPII binding patterns depending on the genomic region. Promoter regions with decreased accessibility were highly enriched with non-phosphorylated Pol II (Non-P) compared with serine 5 phosphorylated Pol II (Ser5P; Fig. 2B). In contrast, genic and intergenic regions with decreased accessibility were largely depleted of Ser5P, while some regions retained Non-P RNAPII binding. There were also distinct differences in RNAPII profiles between the regions that increased compared with those that decreased accessibility (Fig. 2C). At promoters and genic regions with decreased accessibility, RNAPII signal shows a bimodal profile, in contrast to the sharp peaks observed at regions with increased accessibility.

Because RNAPII is enriched at DOCRs, we next determined the level of transcription from these regions (Supplementary Fig. S2). We found most regions (∼90%) had detectable RNA synthesis based on RNA-seq read counts in the region spanning ± 250 bp from the center of each DOCR (Supplementary Fig. S2A). RNA counts showed small increases in expression at regions with increased accessibility, while intergenic regions with decreased accessibility had substantial decreases.

To evaluate further how the changes in accessibility affected transcription initiation, we performed start-seq to assess enrichment of transcription initiation start sites at DOCR classes. We defined the TSSs as either being associated with a known gene (genic), or not associated with a known gene (non-genic), then associated each class of TSSs with GAIN or LOST DOCRs.

**FIGURE 3:**
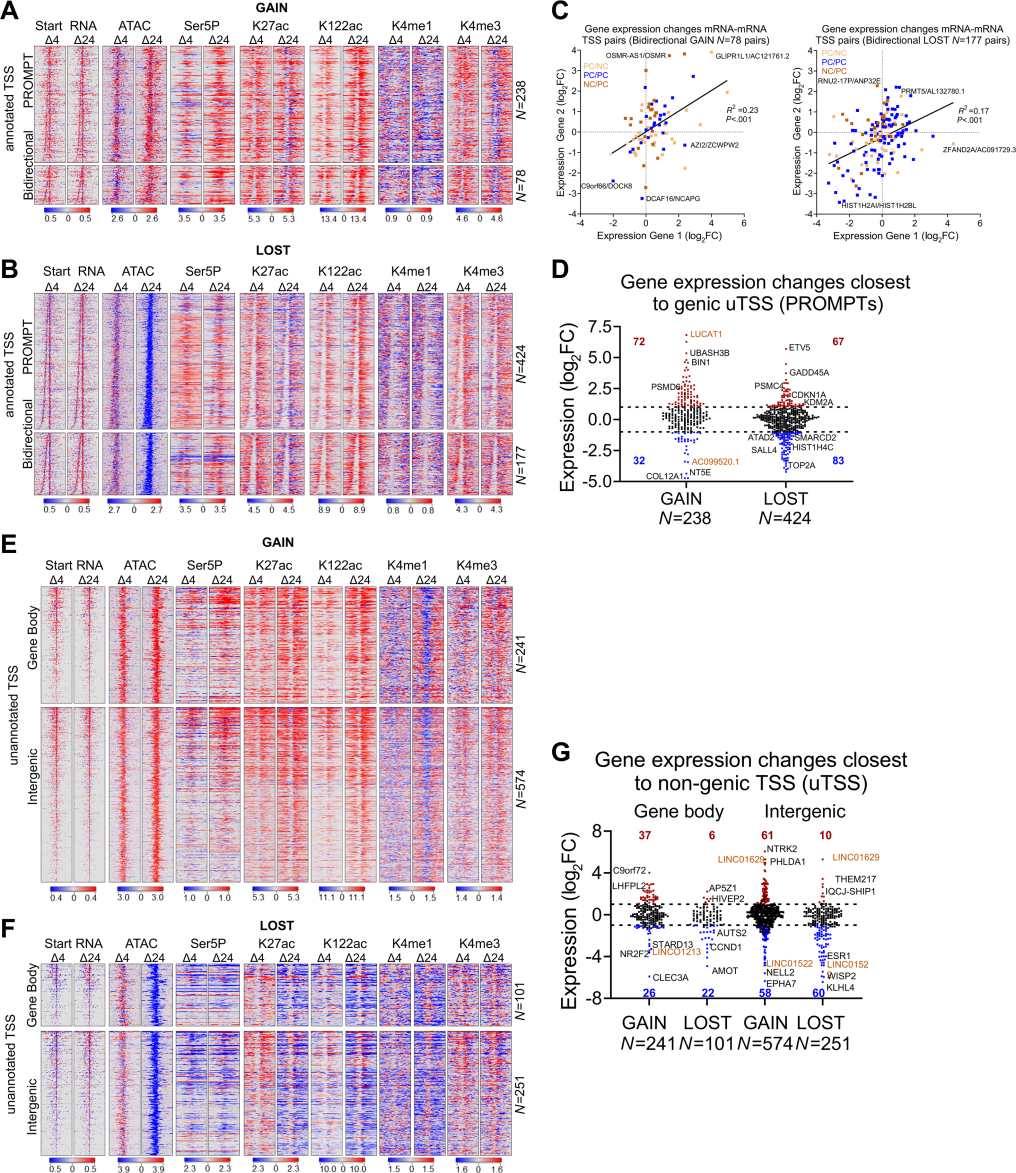
Proteasome inhibition exhibits specific effects on divergent transcription. **A,** Heat maps showing differential signal for start RNA, DNA accessibility (ATAC), RNAPII (Ser5P), and active histone marks (K27ac, K122ac, K4me1, K4me3) at genic TSSs with divergent transcription that overlap **GAIN** DOCRs. **B,** Heat maps as in A, overlap **LOST** DOCRs. Signal spans ±500 bases from the TSSs and is ranked by distance between sense and antisense TSS pairs, where TSSs at the top have the shortest distance between pairs. TSSs are split by category into Promoter Upstream Transcripts (PROMPT) and Bidirectional (head-to-head) TSS pairs. *N* is the number of TSS pairs in each category. **C,** Scatter plot showing gene expression changes of bidirectional (head-to-head) TSS pairs. TSS pairs are colored by gene class, where each TSS in the pair is either protein coding (PC) or non-coding (NC). **D,** Violin plot showing gene expression changes of genic TSSs associated with promoter upstream transcript (PROMPT) category. Significantly different genes (FDR ≤ 0.05, log_2_FC±1) are colored red (upregulated) and blue (downregulated). Gold labeled are non-coding genes. **E,** Heat maps showing differential signal of features in A at non-genic TSSs that overlap **GAIN** DOCRs. **F,** Heat maps showing differential signal of features in A at non-genic TSSs that overlap **LOST** DOCRs. TSSs are split by genomic category into gene body and intergenic. **G,** Violin plot showing gene expression changes of genes closest to non-genic TSSs, which are split by genomic category into gene body and intergenic. *N* is the number of TSS pairs in each category.

We found 316 and 601 genic TSSs that overlapped with GAIN or LOST DOCRs, respectively. Because these primarily represent start sites from promoter regions, which we found to differ in chromatin architecture, we tested whether there were differences in patterns of transcription initiation (Supplementary Fig. S2B; Supplementary Table S3). For this, we first identified genic TSSs that were paired with an upstream divergent TSS. Then we subgrouped divergent genic TSSs based upon whether its pair was a genic bidirectional mRNA-mRNA gene pair, or a non-genic promoter upstream transcript (PROMPT). We found 70% of the divergent transcription from promoters that increase (GAIN), and decrease (LOST) accessibility was due to PROMPTs, whereas 30% was due to bidirectional (head-head) mRNA gene pairs (Fig. 3A and B).

To explore this further, we sorted the PROMPTs and head-to-head pairs based on genomic distance between the sense and antisense TSS pairs, and evaluated the relevant start-seq, RNAPII binding, and select histone modifications. This analysis revealed a striking contrast in the distance between divergent TSSs from DOCRs GAIN compared with LOST (Fig. 3A and B, start-RNA). The median distance between the aggregate TSS pairs was wider in regions of decreased accessibility (277 nt) versus increased accessibility (188 nt) [(*P* < 0.0001; Supplementary Fig. S2C)] and this difference in divergent distance persisted when we stratified TSS into PROMPTs and head-to-head pairs (Supplementary Fig. S2D). The difference in the divergent distance of GAIN and LOST, was evident on heat map profiles of the start-RNA sorted by increasing distance between TSS pairs. RNAPII (Ser5P) and ChIP signals for active histone marks around the TSS pairs reflected the differences in transcription initiation patterns at promoter DOCR classes (Fig. 3A and B). Indeed, apart from initiation patterns, GAIN regions are progressively enriched with marks of active transcription (Ser5P, K27/K122ac, K4me3), whereas LOST regions acquire more of a poised transcriptional state with decreased RNAPII and K27ac which would affect gene expression.

We next assessed transcriptional effects by RNA-seq to test the effect of transcription initiation patterns on gene expression. Gene expression of bidirectional gene pair tended to correlate, regardless of the change in chromatin accessibility (*r*^2^ = 0.23 and *r*^2^ = 0.17). A subset, of these genes (46 and 108) were differentially expressed [36 upregulated vs. 10 downregulated and 40 upregulated vs. 68 downregulated (FDR <0.05; log_2_FC = 1; Fig. 3C; Supplementary Table S3)]. In DOCRs GAIN, more than 65% of the bidirectional genes were composed of protein coding and non-coding pairs, whereas only 42% of the pairs were protein coding/non-coding in the DOCRs LOST. Upregulated gene pairs included signaling molecules (e.g., GLIPR1L1, OSMR) and chromatin regulators (e.g., KDM2A, ANP32E) whereas downregulated genes were predominantly histone H2A and H2B transcripts and chromatin remodelers (ATAD2, SMARCD2). Some examples are highlighted (Fig. 3C; Supplementary Table S3).

We next evaluated how transcription upstream of promoters affected gene expression. Genes associated with GAIN PROMPTs tended to be induced (72 upregulated and 32 downregulated), while genes associated with LOST were slightly repressed (67 upregulated and 83 downregulated) [(FDR <0.05, log_2_FC = 1; Fig. 3D; Supplementary Table S3)]. Examples of induced and repressed genes for both classes of accessible regions are highlighted (Fig. 3D) and browser tracks present chromatin state and transcription initiation sites associated with examples of genes expressed from each class of TSS pairs (Supplementary Fig. S2E). Thus, we conclude proteasome inhibition alters chromatin state and this has specific effects on transcription initiation and gene expression patterns.

### Unannotated Transcription Initiation Near DOCRs is Largely Non-coding

We also explored non-genic TSSs that overlapped with DOCRs (Fig. 3E and F; Supplementary Fig. S3; Supplementary Table S4). We found more non-genic TSSs in regions that increase (815) compared with those that decrease (352) accessibility (Supplementary Fig. S3A; Supplementary Table S4), which we subsequently divided into TSS sites initiating from either gene body or intergenic regions (Fig. 3E and F). Over 70% of non-genic TSSs were intergenic and 30% were in the gene body in regions of increased (GAIN) and decreased (LOST) accessibility, respectively.

Having characterized transcription events occurring at promoter regions affected by MG132, we next explored transcription occurring at regions distal to TSSs. As shown on the heatmaps, non-genic regions with increased accessibility were enriched with Ser5-P, H3K4me1, H3K27ac, and H3K122ac, features depleted in regions with decreased accessibility, inferring actively transcribed *cis*-regulatory elements (Fig. 3E and F). The degree of acetylation and chromatin accessibility in the non-coding regions was associated with aberrant activation or repression of unannotated promoters as shown by start RNA expression (Fig. 3E and F).

We explored whether non-genic RNAPII initiation had any effect on gene expression changes of nearby genes (Fig. 3G). Regions with increased accessibility had a relatively similar number of upregulated and downregulated genes nearby unannotated TSS, whereas regions with decreased accessibility had a substantially higher percentage of downregulated genes. Furthermore, there was no correlation between the expression of the unannotated TSS RNA and the closest gene (Supplementary Fig. S3B). Some examples of induced and repressed genes closest to non-genic TSSs in both classes of accessible regions are highlighted (Fig. 3G; Supplementary Table S4).

Finally, analysis of the start-seq data revealed treatment with MG132 led to additional transcription initiation from genic and intergenic regions, resulting in expression of non-coding RNAs, most of which were uncharacterized novel transcripts whose expression was not detected by RNA-seq (Supplementary Fig. S3C), and those detected were generally repressed (Fig. 3G; Supplementary Table S4). Browser shots show representative examples of GAIN and LOST genic and intergenic regions and their associated unannotated transcription initiation sites (uTSS; Supplementary Fig. S3D).

### Differential Accessibility Affects TF Motifs Related to Chromatin Dynamics and Cell Fate Decisions

In general, sequence specific TFs bind *cis*-regulatory DNA elements within accessible chromatin to orchestrate RNAPII transcription. We applied motif discovery to search for candidate TF motifs that were enriched at DOCRs that displayed increased and decreased accessibility.

**FIGURE 4:**
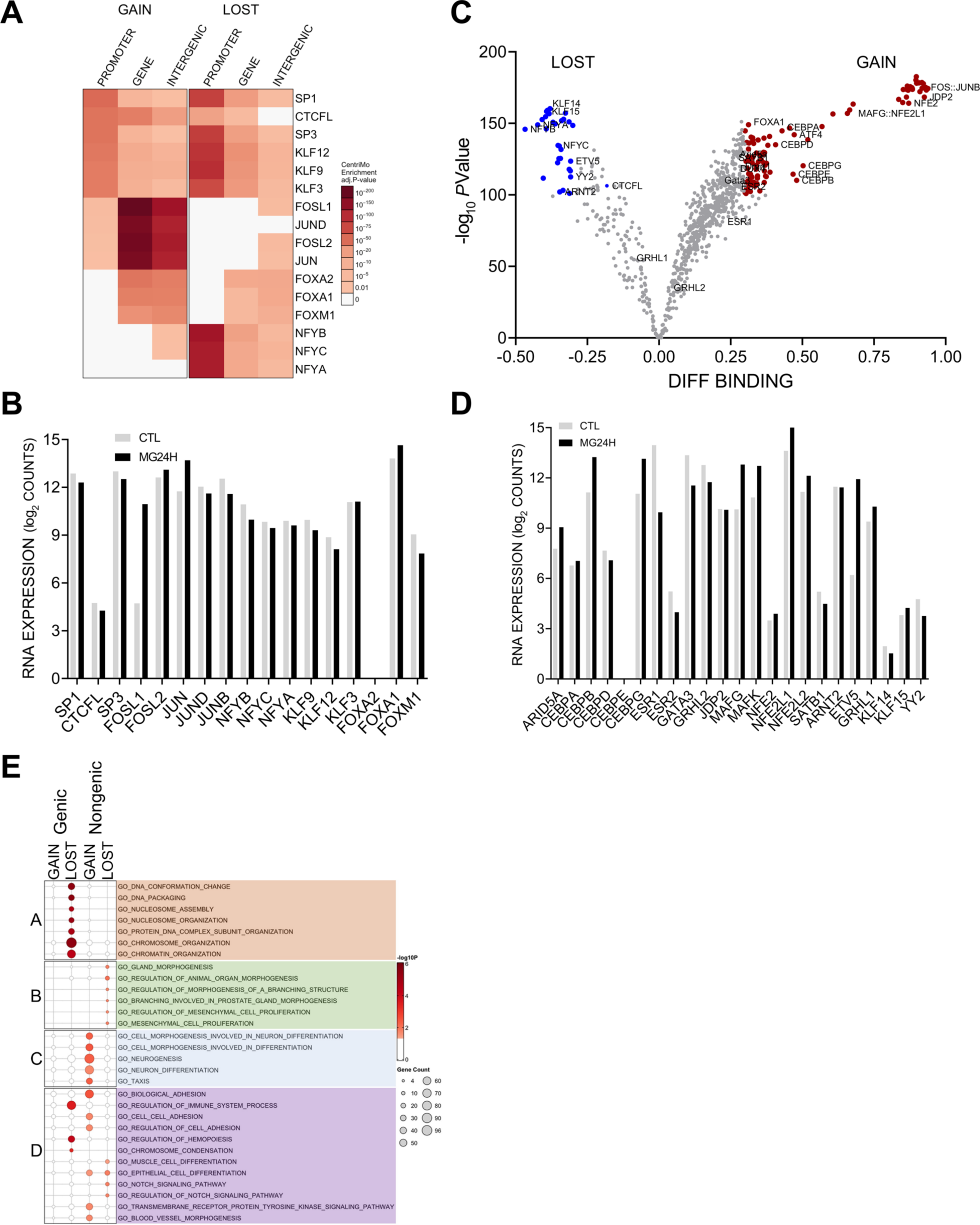
DOCR-associated TF motifs involve chromatin dynamics and cell fate. **A,** Heat map showing the degree of enrichment for top TFs binding motifs identified as enriched in each DOCR category. Each row corresponds to a TF. Each column corresponds to the DOCR genomic category and the heat map is split into DOCR class, **Left** (GAIN) and **Right** (LOST). **B,** Relative mRNA expression of TFs enriched at DOCRs, showing RNA-seq read counts in control (CTL) and 24H treated cells. **C,** Volcano plot showing the degree of differential binding and the statistical significance of the difference for all TF motifs queried. TF motifs with significant differential binding are colored **red** (increase) and **blue** (decrease). **D,** Relative mRNA expression of TFs identified by TOBIAS, showing RNA-seq read counts in control (CTL) and 24H treated cells. **E,** Dot plot showing the degree of enrichment for gene lists enriched with genes closest to DOCRs split by category. Dot size represents the number of genes in each Gene Ontology (GO) biological processes.

We found distinct classes of TF motifs enriched in regions that gained accessibility compared with regions that are less accessible in MG132-treated cells (Fig. 4A). AP-1 (FOS/JUN) motifs were notably enriched in genic and intergenic regions that gained accessibility compared with regions that were less accessible. On the other hand, NFY motifs were specifically enriched at less accessible promoters. Motifs for specificity proteins (SP) and Krüppel-like transcription factors (KLF) were enriched at all regions, with the greatest enrichment at promoters with decreased accessibility. Finally, Fork head transcription factor (FOX) motifs were enriched in the genic and intergenic regions, whereas CTCFL (BORIS/CTCF like) motifs were enriched overall but depleted at intergenic regions with decreased accessibility. Transcripts of most TFs associated with significant motifs were expressed in MCF-7 cells (Fig. 4B). Foot printing analysis of DNA sequences underlying the 14,008 global OCRs using TOBIAS (46) found 267 enriched and 54 depleted motifs, upon 24-hour treatment with MG132 [(*P*value ≤ 10^−100^ cutoff), Fig. 4C)]. These analyses confirmed enrichment of the FOS-JUN family of motifs, and depletion of KLF and NFY motifs. TOBIAS also detected additional TF motifs of interest, including enrichment of NFE2 (NFE2L1/2), ATF, MAF, BACH, CEBP, depletion of YY, ARNT, ETS Variant TFs (ETV5, etc.) and potential repressors, including ARID3B/5A, JDP2, SAT1B, which were also largely expressed in MCF-7 cells (Fig. 4D).

Next, we tested for enrichment of Gene Ontology biological processes in the genes nearest to the DOCRs. DOCRs with decreased accessibility in promoter regions were enriched in chromatin organization, nucleosome assembly, and DNA packaging (Fig. 4E, cluster A). Most genes encoding histones, histone chaperones, and chromatin regulators also appear in cluster A of the concept network (cNET; Supplementary Fig. S4A). Genes in promoter regions with increased accessibility showed no significant enrichment, perhaps because a majority of the TSS code for non-coding RNAs (Fig. 3C).

Genes whose TSS were nearby distal DOCR regions with increased accessibility were enriched for processes involved in neurogenesis (cluster C) and cancer-related processes (cluster D) including cell adhesion. On the other hand, genes in regions of decreased accessibility showed enrichment in processes involved in cell fate and cancer, including gland morphogenesis (cluster B), cell proliferation, and Notch signaling (cluster D). Genes shared by cluster A and D represent a link between chromatin organization and cancer hallmark processes, involving FOXA1, ESRI, PHF14 and the ATPase proteasome subunits, PSMC5 and PSMC4 (Supplementary Fig. S4A).

A follow-up analysis revealed that DOCR-linked genes were specifically enriched in a few Hallmark pathways encompassing a myriad of pathologic processes that may impact breast cancer pathophysiology: mTORC1, Hedgehog signaling, and estrogen response, controlling cell proliferation; E2F regulation control of cell cycle and replication; IL2/STAT5 and ROS signaling involved in ferroptosis cell death and immune response, (Supplementary Fig. S4B). Representative genes of each node are shown in the cNET plot (Supplementary Fig. S4C).

Finally, we utilized the Gene Transcription Regulation Database (GTRD; http://gtrd.biouml.org/) to explore additional transcriptional regulators of DOCR genes (47). We find enriched targets of the histone lysine methyltransferase ASH1L, the TF CEBPZ, and PRKDC (DNA-PK) associated with genes in regions with decreased accessibility (Supplementary Fig. S4D and S4E). On the other hand, regions with increased accessibility represent targets of neurogenesis regulators HES2, PAX3, CBX5, PRDM5, and chemoresistance factor MED16 (Fig. 4D; Supplementary Fig. S4E).

Taken together, after 24-hour proteasome inhibition, we found increased accessibility in genes associated with neurogenesis, and decreased accessibility in genes associated with chromatin maintenance and cell cycle regulation.

### Proteasome Effects on Accessibility Stratify Non-basal Breast Cancer Tumor Subtype

**FIGURE 5:**
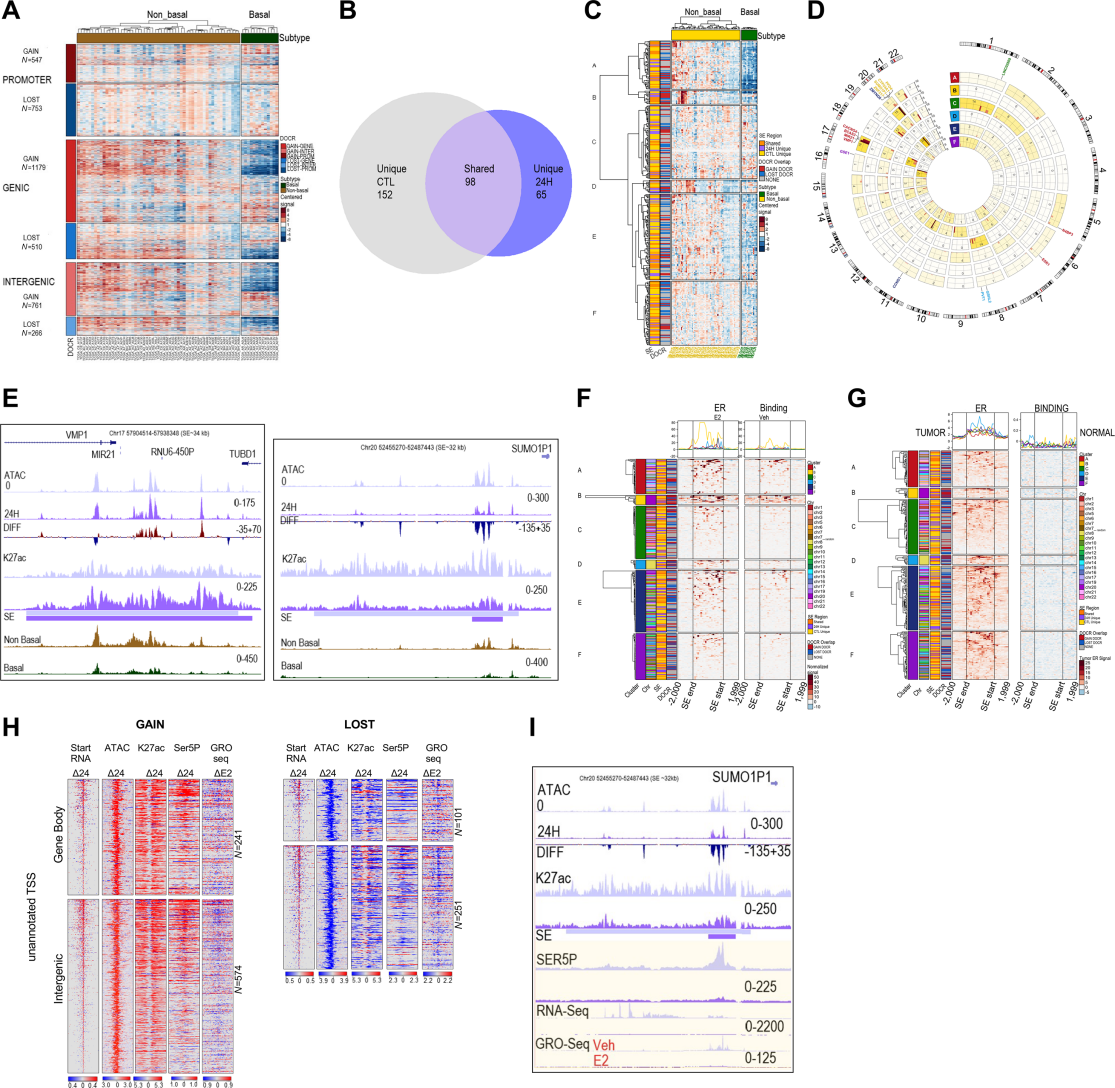
DOCRs define important *cis*-regulatory regions specific to non-basal breast cancer tumors. **A,** Heat map showing chromatin accessibility signal (ATAC-seq) of TCGA breast cancer tumors (39) in DOCRs. Each row corresponds to a DOCR, and they are split by increase (**GAIN**) or decrease (**LOST**) in accessibility and by genomic category (**PROMOTER**, **GENIC**, **INTERGENIC**). *N* is the number of DOCRs in each category. Each column corresponds to a TCGA breast cancer tumor, split by non-basal (*N* = 57) and basal (*N* = 13) subtypes. Signal is z-score normalized by row, where red is high relative signal and blue is low relative signal. **B,** Venn diagram highlighting the number of SEs identified in Control only, 24H treated only, or shared between the samples. **C,** Heat map showing chromatin accessibility signal (ATAC-seq) of TCGA breast cancer tumors in SEs. Each row (*N* = 333) corresponds to a SE region, and they are split by cluster from hierarchical clustering. **D,** Circos plot showing chromosome coordinates of SEs, split by cluster. The outer ring shows reference chromosomes 1 through 22 in clockwise orientation. Inside rings correspond to clusters A–F from the heat map in C. The number of SE regions in each cluster per chromosome is indicated inset. Representative genes in SE regions in each cluster are shown. Color density indicates SE enrichment within each cluster and chromosome. **E,** Browser tracks showing the VMP1/MIR21 and SUMO1P1 SE regions. Tracks show read coverage of chromatin accessibility (ATAC), differential read coverage of accessibility (DIFF), H3K27ac read coverage, SE regions, and average ATAC read coverage for non-basal (brown) and basal (green) TCGA breast cancer tumors. Each track represents a control (0) light and 24H sample dark purple. **F,** Heat map showing ER ChIP-seq signal of MCF-7 cells treated with E2 (left) or Vehicle (right) in SE regions. Signal spans from 2 kb upstream of the SE, through the body of the SE, and to 2 kb downstream of the SE. Rows are split by cluster from the heat map in C. Signal is z-score normalized by row, where red is high relative signal and blue is low relative signal. **G,** Heat map showing ER ChIP-seq signal of breast tumor (left) or normal tissue (right) in SE regions. **H,** Heat maps showing differential signal for start RNA, accessibility (ATAC), RNAPII (Ser5P), K27ac and GRO-seq (E2) at non-genic TSSs that overlap **GAIN** (left) and **LOST** DOCRs (right). **I,** Browser tracks showing the SUMO1P1 SE region. Tracks show read coverage of chromatin accessibility (ATAC), differential accessibility (DIFF), SE regions, K27ac, RNAPII (SER5P), RNA-seq, and GRO-seq from cells treated with Veh and E2.

We evaluated the accessibility of MG132 DOCR regions in breast cancer tumors by leveraging data from ref. 39, where they analyzed chromatin accessibility in primary human cancers including breast cancer. We plotted accessibility across breast cancer samples for DOCRs, subdivided by promoter, genic, and intergenic regions (Fig. 5A). The hierarchical clustering of open chromatin signal within DOCRs clearly separated non-basal (hormone receptor/ER positive) from basal (hormone receptor negative) breast cancer subtypes, with differences more substantial in genic and intergenic compared with promoter DOCRs (Fig. 5A).

We next defined SEs using ROSE, which identified 250 in control and 164 in MG132-treated cells (Supplementary Fig. S5A; Supplementary Table S5). Among these, 98 SEs were shared between control and MG132-treated cells, and most of them (82 SEs, 84%) overlapped with at least one DOCR (Fig. 5B; Supplementary Table S5). Most (∼65%) SEs unique to control cells did not overlap DOCRs, whereas SEs unique to MG132-treated cells showed higher overlap with DOCRs, and these were primarily in regions that increased chromatin accessibility (Fig. 5B; Supplementary Table S5). In addition, SEs unique to MG132 treatment had lower H3K27ac signal compared with the shared SEs (Supplementary Fig. S5B). Representative examples of each class of SE are shown (Supplementary Fig. S5C).

On the basis of this analysis, we sought to understand the biological significance of the SEs in breast cancer biology. We examined TCGA breast cancer ATAC-seq signal at the SE regions which overlap OCRs (Fig. 5C; Supplementary Table S6). Hierarchical clustering was used to split the SEs into six subclusters with distinct DNA accessibility profiles. Cluster A contained 54 SEs which showed substantially lower accessibility in basal compared with non-basal breast tumors. Interestingly, 16 cluster A SEs were located on chromosome 17 (Fig. 5D). One such SE which overlaps the VMP1/MIR21 gene locus showed increased accessibility with MG132 but decreased accessibility in basal tumors (Fig. 5E; Supplementary Fig. S5D). Other SEs of interest in cluster A were in regions that show decreased accessibility in treated cells, including an SE in the BCAS3 intron, an SE in nearby of CACNG4, a region in the proximity (∼13 kb) of N4BP3 (Chr 5) and ESR1 (Chr 6; Fig. 5D, highlighted; Supplementary Table S6).

Clusters B and D consisted of 15 and 14 SE regions, respectively, and showed high accessibility in a subset of non-basal breast tumors. All cluster B SE regions were located on chromosome 20, and all cluster D regions were on chromosome 8 (Fig. 5C and D). While accessibility in basal regions of both clusters B and D was low, the degree of accessibility in cluster B regions on chromosome 20 was even lower than cluster D. The majority of cluster B SEs (14/15) overlapped with DOCRs, and 10 were in regions of increased DNA accessibility. The regions that decreased chromatin accessibility are in Chr 20 gene deserts, neighboring non-coding RNAs. One such region is near the SUMO1P1 pseudogene and shows a dramatic decrease in accessibility in MG132-treated cells, along with high accessibility in non-basal breast tumors (Fig. 5E, right). Furthermore, the SUMO1P1 desert region is more accessible in highly proliferative compared with lowly proliferative breast tumors (Supplementary Fig. S5D). Cluster D SEs also largely overlapped DOCRs (11/14) and included SEs nearby PVT1 and GRHL2 (Fig. 5D; Supplementary Table S6).

Cluster C, E, and F SEs showed modest changes in accessibility, but differences across breast tumors were still evident (Fig. 5C).

Summary figures for SE regions near VMP1/MIR21 (Chr 17), SUMO1P1 (Chr 20), and PVT1 (Chr 8) are shown as representative examples from cluster A, B, and D, respectively, showing higher accessibility in non-basal compared with basal breast tumor samples (Supplementary Fig. S5D). Figures depicting SE regions near LINC02869 (Chr1), ZMYND8 (Chr 20), and GSE1 (Chr 16) are shown as representative examples of clusters C, E, and F, respectively (Supplementary Fig. S5E).

Non-basal breast tumors are characterized by the expression of ER, so we investigated whether the SE regions were associated with ER binding sites (Fig. 5F and G). To this end, we downloaded publicly available ChIP-seq data for ER in MCF-7 cells and breast tumor samples (41) to examine ER binding at SE regions. We found enriched ER binding in MCF-7 cells concentrated in clusters A and B, which were clusters highly accessible in non-basal compared with basal tumors (Fig. 5F). We also found these regions were enriched with ER in ER-positive tumors compared with normal breast tissue (Fig. 5G).

Cancer cells are highly dependent on an active proteasome to modulate gene regulatory networks that drive cell growth, but an important question is whether MG132 represses aberrant transcription associated with ER-dependent transcription. DOCRs in the gene body and intergenic regions are transcriptionally active in MG132-treated cells (Fig. 3E and F). Next we leveraged data from GRO-seq (48) of ER regulated transcription in MCF-7 cells to examine whether transcription at these regions was sensitive to E2 treatment. We observed transcriptional induction and repression at sites with both increased and decreased accessibility, respectively upon E2 treatment as shown on heat maps of GRO-seq signal detected across non-genic TSS overlapping DOCRs in cells treated with vehicle or estradiol (E2; Fig. 5H; Supplementary Fig. S6A) We then focused on a region in cluster B containing intergenic DOCRs with decreased accessibility on chromosome 20 (chr20:52462606-52487433), which also includes a large SE that overlaps DOCRs close to SUMO1P1 and shows increased ATAC signal in proliferative non-basal tumors (Fig. 5I; Supplementary Fig. S5D, center). In MG132-treated cells, the region showed a consistent decrease in accessibility, RNAPII binding, and RNA transcription (Fig. 5I). On the basis of GRO-seq, compared with vehicle (veh), E2 treatment also repressed transcription from this region. The decrease in RNA expression seen in the RNA and GRO-seq was confirmed by qPCR using primers spanning the SE region (Supplementary Fig. S6B). The effects of MG132, relative to E2, were further validated using known ER targets TFF1and ZNF217 (Supplementary Fig. S6C).

These data indicate that proper proteasome function is important for breast cancer biology, as proteasome inhibition partly disrupts ER-dependent transcriptional programs that regulate cellular processes critical for cell growth.

## Discussion

Inhibiting proteasome activity is widely believed to disrupt RNAPII transcription initiation, ceasing global gene transcription and expression, although accompanying chromatin changes leading to these events is largely speculative and unexplored (7). Using a low dose of the small molecule MG132 enables investigation of the effect of proteasome inhibition on chromatin accessibility and RNAPII transcription in breast cancer cells. Promoter DNA accessibility is very sensitive to proteasome inhibition, of 50% of the regions that decrease accessibility are in promoter regions, agreeing with existing data indicating proteasome activity is required for turnover and recycling of RNAPII preinitiation complexes to allow multiple rounds of transcription (reviewed in refs. 10, 49). Thus, stalled or poised ubiquitinated RNAPII, evidenced by enriched non-phosphorylated Pol II at these promoters, could result in the observed decrease in accessibility (32, 50). In addition, the decrease in accessibility is a result of decreased histone acetylation, or eviction of other histone marks generally enriched at promoters, such H2A.Z (51). Interestingly, a subset of promoters does not require proteasome activity to open and this correlates with acetylated H3K27ac, and H3K122ac together with transcriptionally active RNAPII-Ser5-P whose binding is coincident with accessible chromatin (52–54).

A major finding from this study is the observed distinct differences in the shape of the GAIN and LOST promoters, which present as unimodal and bimodal profiles of histone marks and RNAPII, respectively. Previous studies have shown bimodal-shaped gene promoter peaks present large nucleosome-depleted regions which are associated with widespread divergent transcription (43, 55, 56). Our study presents additional evidence to show bimodal and unimodal promoters exhibit distinct types of divergent transcription patterns in cells treated with proteasome inhibitor MG132. Taking advantage of high-resolution strand-specific mapping of TSSs, we stratify divergent transcription into head-head (mRNA-mRNA) and promoter upstream antisense transcription (PROMPT), to show divergent transcription initiation patterns differ between the two types of promoters, resulting in transcription of distinct classes of genes. Strikingly, at bimodal promoters, bidirectional head-head transcription initiation was predominantly from histone gene cluster TSS pairs, whereas PROMPT-mRNA transcription significantly encoded chromatin regulators and histone chaperones, an observation consistent with a functional role of divergent transcription in the regulation of specific classes of genes in the human genome (57–59). In contrast to bimodal, unimodal promoters, head-head, and PROMPT divergent TSS pairs included a combination of long non-coding RNAs, and protein coding genes not typically expressed in breast cancer cells. These genes were enriched in neural pathways including neurogenesis (60, 61). This finding is consistent with a crucial role for the proteasome in preventing aberrant transcription initiation to maintain transcription fidelity and cell/tissue specific gene expression (reviewed in ref. 10; ref. 62).

The effects of proteasome inhibition on chromatin accessibility were not limited to promoters. We observed large changes in DNA accessibility at regions distal to TSS which were enriched or depleted of enhancer histone marks and RNAPII-Ser5P, suggesting inhibiting proteasome activity reprograms the enhancer landscape of breast cancer cells. On the basis of the RNAPII density, the reprogrammed enhancers were functionally active as we detected increased and decreased start RNAs in regions where changes in accessibility are observed. Importantly, in contrast to promoters, which were less accessible, distal regions were hyperacetylated, and more open, resulting in spurious transcription of non-coding RNAs as detected by start-seq. These observations reinforce the notion that the proteasome is required for transcriptome integrity, and inhibiting its function results in pervasive transcription, which can result in dysregulation of gene networks that may influence cell fate decisions (62–64).

Indeed, changes in accessibility of the enhancer landscape occur at SE regions that regulate transcription and expression of gene networks associated with cell proliferation and chemoresistance, cell fate decisions critical for breast cancer tumorigenesis (65–68). A subset of the SE regions is predominantly accessible in non-basal compared with basal breast cancer subtypes. Of note these regions are primarily in chromosomes 8, 17, and 20 hot spot loci, characterized by genomic amplification, aneuploidy, oncogene translocations, and genomic alterations that result in oncogene activation in breast cancer (69–71). Our findings reveal a yet uncharacterized role for the proteasome in regulating the accessibility and activity of *cis*-regulatory elements, important in breast cancer biology (39, 72–74).

Breast cancer subtype classification is generally based on hormone receptor status, where the non-basal and basal subtypes are ER positive or negative, respectively (75). ERs are critical regulators of breast epithelial cell proliferation, a major factor contributing to breast cancer tumorigenesis (76). Gene expression networks controlling cell proliferation were repressed by MG132 treatment supporting proteolytic function of the proteasome in ER-mediated transcriptional activity (24). Concomitantly, here we showed inhibiting proteasome function impacted accessibility of ER bound SE regions in hormone receptor–positive breast cancers. Estrogen receptors are ligand-activated TFs, whose turnover and transcriptional activity is tightly controlled by the proteasome (reviewed in refs. 77, 78). Our findings suggest MG132 may affect accessibility of ER-enriched SEs, important in the regulation of ER signaling pathways implicated in breast cancer biology.

Finally, our study points to chromatin accessibility as a potential mechanism by which proteasome inhibitor drugs exert their anticancer effects. We showed dramatic changes in accessibility and transcription, at a subset of SE regions that distinguish non-basal and basal breast cancer subtypes and are also implicated in regulating expression of genes related to cell proliferation and chemoresistance (39, 72–74, 79). In addition, MG132 suppresses ER-enriched SE transcription, suggesting proteasome inhibition alters the function of a critical TF involved in the biology of hormone receptor–positive breast cancers. SEs play vital roles in tumorigenesis and small-molecule drugs targeting SE function and the molecular machinery that maintain their chromatin state and transcriptional activation represent promising therapeutic strategy for cancer treatment (19, 80, 81). Thus, modulating SE accessibility and transcription is a potential mechanism by which proteasome inhibitor pharmaceuticals could exert anticancer effects in solid tumors, such as breast cancer. Such a mechanism has been suggested by a recent study showing proteasome inhibition caused H3K27 deacetylation of a SE in control of transcription of the c-MYC proto-oncogene, which resulted in decreased growth of multiple myeloma cells, a blood cancer where proteasome inhibitors are primarily used as therapy (82). Furthermore, other small-molecule chemotherapeutics have been shown to disrupt chromatin accessibility and RNAPII transcription of critical *cis*-regulatory elements as potential mechanisms for controlling gene expression networks related to cancer cell death (83, 84).

We have shown treatment of MCF-7 breast cancer cells with the proteasome inhibitor MG132 results in differential changes in chromatin accessibility and RNAPII transcription. These accessibility changes were discrete such that losses were largely at promoters, and gains were predominantly at regions distal to TSS. Promoters which were affected by MG132, displayed diverse chromatin architecture, resulting in divergent transcription of TSS pairs that code for distinct classes of genes, including the HIST1 histone gene cluster, chromatin regulators and novel non-coding RNAs. Chromatin changes also occurred at regions distal to TSS that overlap SE elements accessible in non-basal compared with basal breast cancer tumor subtypes. Our study reveals a yet uncharacterized molecular mechanism by which disruption of proteasome function affects the chromatin landscape, transcription, and expression of gene regulatory networks important in breast cancer biology.

### Limitations of the Study

Our study has some limitations. We only use MG132 as the proteasome inhibitor and MCF-7 cells as the representative breast cancer cell line. However, the use of MG132 as an experimental drug to inhibit proteasome activity is largely accepted in the field (85). Furthermore, the biological effects we observe with MG132 are replicated by other proteasome inhibitor drugs (23). MCF-7 breast cancers are routinely used as a model to study hormone receptor–positive breast cancers (86), and our findings are relevant to breast cancer biology

The genomics approaches taken in this study do not identify specific factors responsible for the differences in chromatin accessibility and RNAPII transcription during proteasome inhibition. Nevertheless, we speculate MG132 affects the expression of machinery that influences chromatin architecture, for example histone genes, their chaperones (e.g., ANP32E), components of epigenetic enzymes (ATAD2, KDM2A, SMARCD2) and non-coding RNAs, all which can have a global impact on chromatin organization, including the observed time-dependent region-specific changes in chromatin accessibility and enhancer reprogramming (65, 87–89). Interestingly, these factors have been implicated in ER-mediated chromatin reorganization and transcriptional regulation. For example, ANP32E (induced by MG132) is a histone chaperone that regulates H2AZ deposition to modulate chromatin accessibility at FOXA1 binding sites and this is inversely associated with tumor growth in ER-positive breast cancers (90, 91). Similarly, the histone demethylase KDM2A (induced) acts as E3 ligase to regulate RNAPII ubiquitination and ER-mediated gene repression, whereas ATAD2 (repressed) regulates p300-mediated histone hyperacetylation to activate ER target genes involved breast cancer biology (92, 93). Finally, SMARCD2 (repressed), is a BRG-1 associated factor, a well-known hormone receptor–mediated chromatin remodeler (94). Altogether, inhibiting proteasome activity can potentially affect the expression of unstable transcripts (e.g., PROMPTs) to regulate the expression of chromatin regulators that modulate accessibility, for example at binding sites of ER and ER cofactors (e.g., FOXA1), thus changing the biology of breast cancer.

In addition to well-characterized chromatin and transcriptional regulators, inhibiting proteasome activity, potentially switches the proteasome from proteolytic to non-proteolytic functions on chromatin. Indeed, we show inhibiting proteasome activity results in a stress-induced negative feedback loop, leading to increased expression of proteasome subunits and particularly the 19S ATPase subunits (24, 95). The 19S ATPase subunits, members of the ATPases associated with various cellular activities family (AAA; ref. 96), are versatile components of chromatin regulators, histone chaperones, and RNAPII complexes, and through this feedback loop mechanism, probably hijack some chromatin remodeling activities when proteolytic activity is inhibited (11–13, 16). In fact, although the majority of the evidence supporting this hypothesis was obtained in yeast (64), in mammalian cells, ATPase subunits bind to hormone receptor gene promoters to modulate chromatin and transcription (32, 97, 98), are enriched at cell type–specific enhancesomes (62, 99), and are components of transcriptionally active condensates and RNAPII complexes (11, 12, 16, 100). Incidentally, this moonlighting activity of the 19S proteasome subunits is not limited to functions on chromatin. A recent study found that the 19S complex was abundant near brain synapses where it regulates synaptic proteins that control excitatory synaptic transmission, independent of the full 26S proteasome complex (101). Interestingly, we show proteasome inhibition, which predominantly increases the expression of 19S subunits, also results in open chromatin at regions that code for genes involved in neurogenesis. Altogether 19S ATPase subunits enrichment at cell type–specific enhancesomes suggests subunit association with chromatin, which could be a potential mechanism for the increase in DNA accessibility observed at distal *cis*-regulatory elements, when proteasome is inhibited.

Time constraints in this study do not allow us to pursue these questions, but future research may be necessary to address these potential limitations.

## Supplementary Material

Figure S1 Shows detailed analysis of changes in DNA accessibility in cells treated with MG132.

Figure S2 Shows effects of proteasome inhibition on different transcription initiation patterns.

Figure S3 Shows effects of proteasome inhibition on non-coding transcription.

Figure S4 Shows biological processes affected by MG132 treatment.

Figure S5 Shows Effects of MG132 on accessibility of breast cancer specific super enhancers and predicted associated genes.

Figure S6 Shows effects of MG132 on estrogen receptor-mediated transcription.

Table S1 List of resources and experimental reagents used in the study.

Table S2 List of genomic coordinates of open (OCR) and differential open chromatin regions (DOCR) in cells treated with MG132, relating to Figure 1.

Table S3 List of genomic coordinates of bidirectional and PROMPT TSS gene pairs and corresponding gene expression changes, relating to Figure 3.

Table S4 List of genomic coordinates of nongenic TSS (uTSS) and nearby gene expression, relating to Figure 3.

Table S5 Genomic coordinates of super enhancer regions, relative to DOCRs identified in control and MG132 treated cells, relating to Figure 5.

Table S6 Genomic coordinates of super enhancer regions per each cluster and nearby genes associated with basal and non-basal breast cancer subtypes, relating to Figure 5.
