# Supplementary material for: Proteasome Inhibition Reprograms Chromatin Landscape in Breast Cancer
Source: Cancer Res Commun. 2024 Apr 16;4(4):1082–99. doi: 10.1158/2767-9764.CRC-23-0476 (PMC11019832; doi:10.1158/2767-9764.CRC-23-0476)
Supplement: Figure S1 — Shows detailed analysis of changes in DNA accessibility in cells treated with MG132. [file crc-23-0476-s01.pdf]

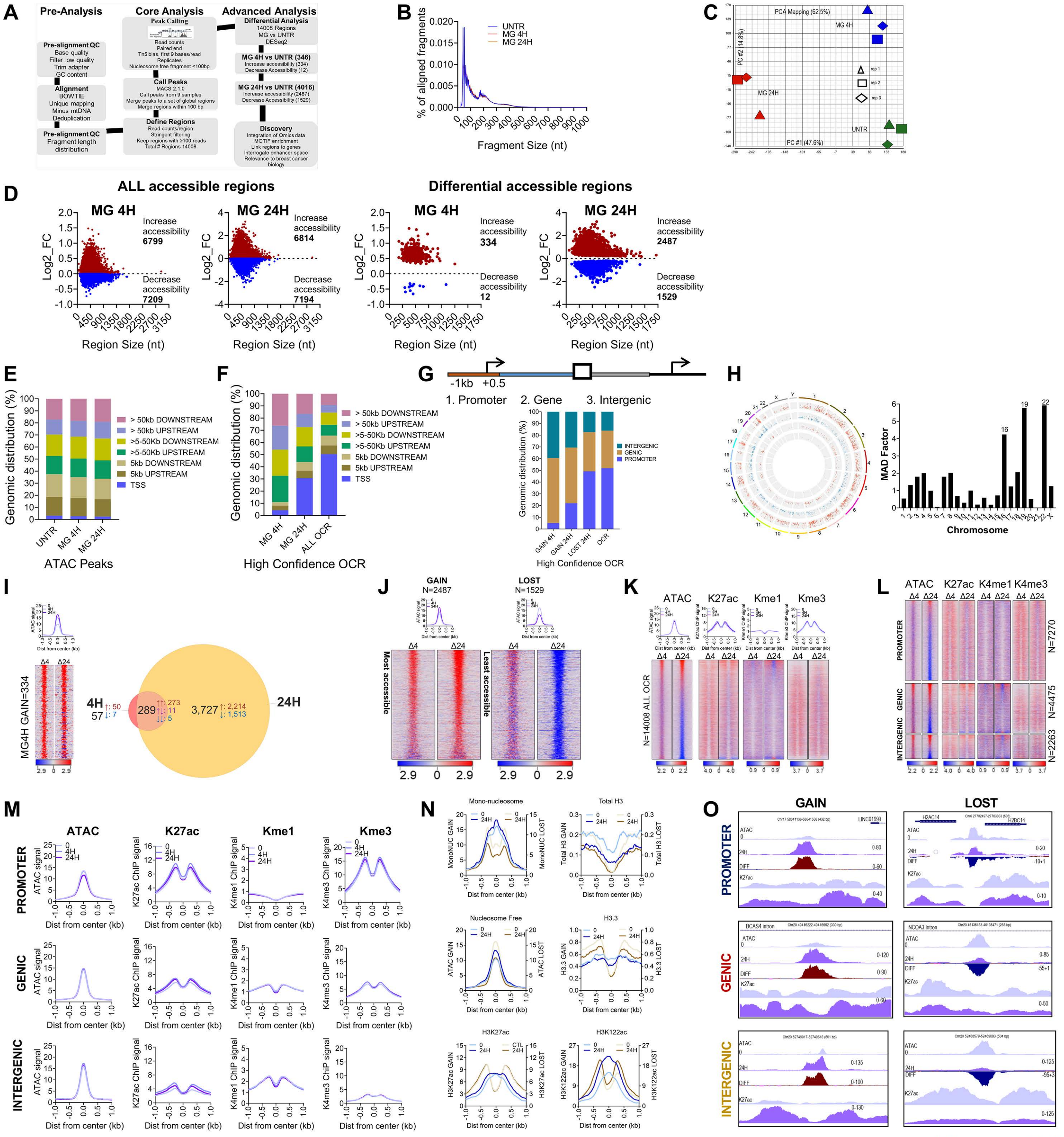

A
Pre-Analysis
Core Analysis
Advanced Analysis
Pre-alignment QC
Alignment
Call Peaks
Define Regions
Differential Analysis
MG 4H vs UNTR (346)
MG 24H vs UNTR (4016)
Discovery
B
% of aligned fragments
Fragment Size (nt)
UNTR
MG 4H
MG 24H
C
PCA Mapping (62.5%)
D
ALL accessible regions
Differential accessible regions
Increase accessibility 6799
Decrease accessibility 7209
Increase accessibility 6814
Decrease accessibility 7194
Increase accessibility 334
Decrease accessibility 12
Increase accessibility 2487
Decrease accessibility 1529
Region Size (nt)
E
ATAC Peaks
F
High Confidence OCR
G
1. Promoter
2. Gene
3. Intergenic
H
MAD Factor
Chromosome
I
J
GAIN N=2487
LOST N=1529
K
L
M
N
O
GAIN
LOST
PROMOTER
GENIC
INTERGENIC

Figure S1

Figure S1 . Systematic analysis of MG132 Differential Open Chromatin Regions (DOCRs) A) Flowchart describing the computational analysis pipeline used to define DOCRs from ATAC-seq. Major steps of analysis are highlighted. **B)** Graph showing insert size distribution of ATAC-seq fragments. **C)** Principal component analysis of ATAC-seq reads showing clear separation among treatment conditions, UNTR (Control, green), MG4H (blue), MG24H (red) are represented on two principal components with PC1 on x-axis and PC2 on y-axis. Three biological replicates per treatment condition are represented (rep1, rep2, rep3). PCA is based on ATAC signal, Tn5 transposase sensitive reads derived from 78, 380 high-confidence regions (FDR 0.05) with at least 10 reads in the 9 samples. **D)** Scatter plots showing the relationship between genomic size of accessible region (nt) and change in chromatin accessibility in MG132 treated samples (log2FC). ALL accessible regions (OCR) 4H (left) and 24H (right) and DOCRs that significantly increase or decrease chromatin accessibility at 4H (left) and 24H (right). **E)** Graph showing genomic distribution of accessible (ATAC) peaks in each treatment condition. **F)** Graph showing genomic distribution of MG132 high confidence open chromatin regions (OCRs) used in the analysis **G)** Graph showing distribution of high confidence OCRs by genomic category. TOP: Schematic defining genomic categories. **H)** Circos plot showing chromosome distribution of MG132 DOCRs. The outer ring represents reference chromosomes 1 through 22 in clockwise orientation. X axis denotes chromosome demarcation, y axis dots are DOCRs ranked by log2FC from 0 to max 2 change in accessibility. From outer circos ring Chromosome, 24H increase (blue), 24H decrease (red), 4H increase (blue), 4H decrease (red). **Right Graph:** Graph showing the Median Absolute Deviation (MAD Factor) indicating variability in DOCR chromosome distribution **I)** Chromatin accessibility of 334 GAIN DOCRs in cells treated for 4H. Heatmaps representing differential ATAC signal (ATAC) and metaplot (Top) representing ATAC signal coverage at genomic regions that change accessibility at 4H, the lines represent signal in control, 0, 4H and 24H treated cells: **Right Panel:** Venn diagram showing the overlap of genomic regions that increase or decrease chromatin accessibility in cells treated for 4 and 24H. Numbers represent DOCRs in each class, arrows represent direction of change in accessibility (red, GAIN), blue, LOST). **J)** Heatmaps and metaplots (top) representing ATAC signal (ATAC) at DOCRs that <u>GAIN</u> (2487, **Right Panel**) and <u>LOST</u> (1529, **Left Panel**). Heatmaps show differential accessibility (ATAC), metaplots show ATAC signal in each treatment sample. **K)** Heatmaps and metaplots (TOP) showing ATAC, H3K27ac, H3K4me1 and H3K4me3 signal at ALL OCR tested (14008) at 0, 4 and 24H. **L)** Heatmaps showing differential ATAC, H3K27ac, H3K4me1 and H3K4me3 signal at ALL OCRs split in genomic categories, PROMOTER (7270), GENE (4475) and INTERGENIC (2263) regions. **M)** Metaplots showing chromatin state for all OCR PROMOTER (7270), GENE (4475) and INTERGENIC (2263). **N)** Metaplots showing distinct chromatin states of GAIN and LOST promoter DOCR. Unique chromatin architecture reflected by the unimodal and bimodal profiles of mono nucleosome signal, ATAC, Total H3, H3.3, H3K27ac, H3K122ac. **O)** Browser tracks showing examples of GAIN and LOST DOCRs. Tracks show read coverage of chromatin accessibility (ATAC), differential read coverage of accessibility (DIFF, GAIN=red, LOST=blue) and H3K27ac Track colors: Control (0), MG24H (24H).
