# Supplementary material for: Proteasome Inhibition Reprograms Chromatin Landscape in Breast Cancer
Source: Cancer Res Commun. 2024 Apr 16;4(4):1082–99. doi: 10.1158/2767-9764.CRC-23-0476 (PMC11019832; doi:10.1158/2767-9764.CRC-23-0476)
Supplement: Figure S2 — Shows effects of proteasome inhibition on different transcription initiation patterns. [file crc-23-0476-s02.pdf]

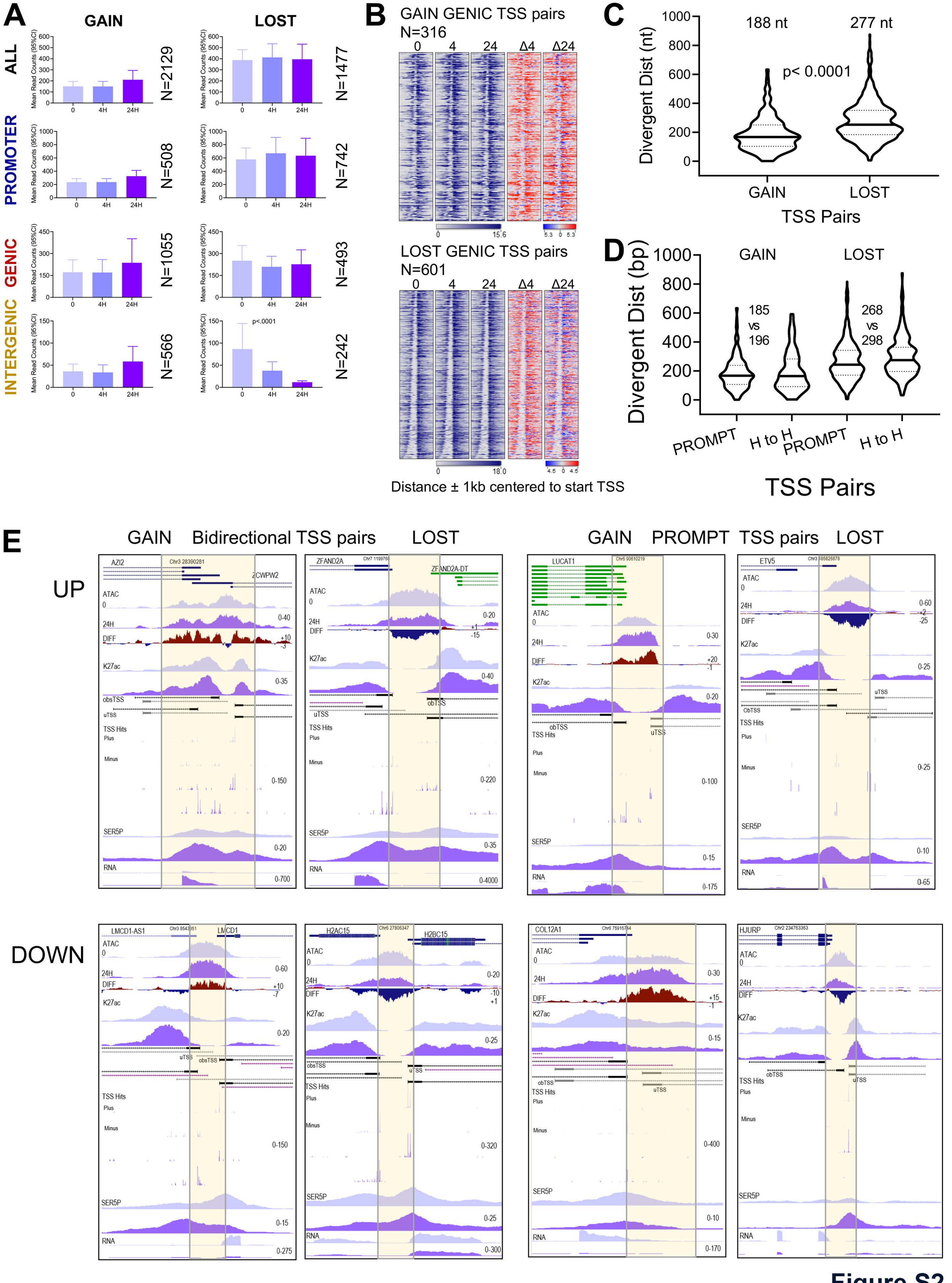

A
GAIN
LOST
ALL
PROMOTER
GENIC
INTERGENIC
Mean Read Counts (95%CI)
N=2129
N=1477
N=508
N=742
N=1055
N=493
N=566
N=242
p<.0001
0
4H
24H
B
GAIN GENIC TSS pairs
N=316
LOST GENIC TSS pairs
N=601
0
4
24
Δ4
Δ24
Distance ± 1kb centered to start TSS
C
188 nt
277 nt
p< 0.0001
Divergent Dist (nt)
GAIN
LOST
TSS Pairs
D
Divergent Dist (bp)
GAIN
LOST
185
vs
196
268
vs
298
PROMPT
H to H
TSS Pairs
E
GAIN
Bidirectional TSS pairs
LOST
PROMPT
TSS pairs
UP
DOWN
AZI2
ZCWPW2
ZFAND2A
ZFAND2A-DT
LUCAT1
ETV5
LMCD1-AS1
LMCD1
H2AC15
H2BC15
COL12A1
HJURP
ATAC
24H
DIFF
K27ac
obsTSS
uTSS
TSS Hits
Plus
Minus
SER5P
RNA

Figure S2

Figure S2. Differential chromatin accessibility dictates distinct transcription initiation patterns A) Graph representing RNA expression (RNA-seq) read counts overlapping DOCRs that significantly increase (GAIN) and decrease (LOST) accessibility. Reads from RNA-seq are within the region +/- 250 bp of the DOCR center. N= DOCR regions with detectable RNA, e.g., for GAIN ALL 2129 of 2487 (90%). **B)** Heatmap showing average H3K27ac signal near genic TSS pairs within GAIN and LOST regions. Signal is ranked based on distance between TSS pairs. N is the number of genic TSS within each DOCR **C)** Violin plots comparing the distance between divergent GAIN and LOST TSS pairs. **D)** Violin plots comparing the distance between bidirectional (head-to-head) vs PROMPT TSS pairs. **E)** Browser tracks showing examples of bidirectional (head-head) and PROMPT TSS pairs around GAIN and LOST DOCRs. Tracks show read coverage of chromatin accessibility (ATAC), differential ATAC (DIFF), H3K27ac, start RNA hits, strand, Ser5P RANPII, RNA expression. Chromosome start coordinate for each TSS is shown. Names of TSS gene pairs are indicated. Track Control (0), MG24H (24H). Box highlights DOCR region and TSS hits.
