# Supplementary material for: Proteasome Inhibition Reprograms Chromatin Landscape in Breast Cancer
Source: Cancer Res Commun. 2024 Apr 16;4(4):1082–99. doi: 10.1158/2767-9764.CRC-23-0476 (PMC11019832; doi:10.1158/2767-9764.CRC-23-0476)
Supplement: Figure S3 — Shows effects of proteasome inhibition on non-coding transcription. [file crc-23-0476-s03.pdf]

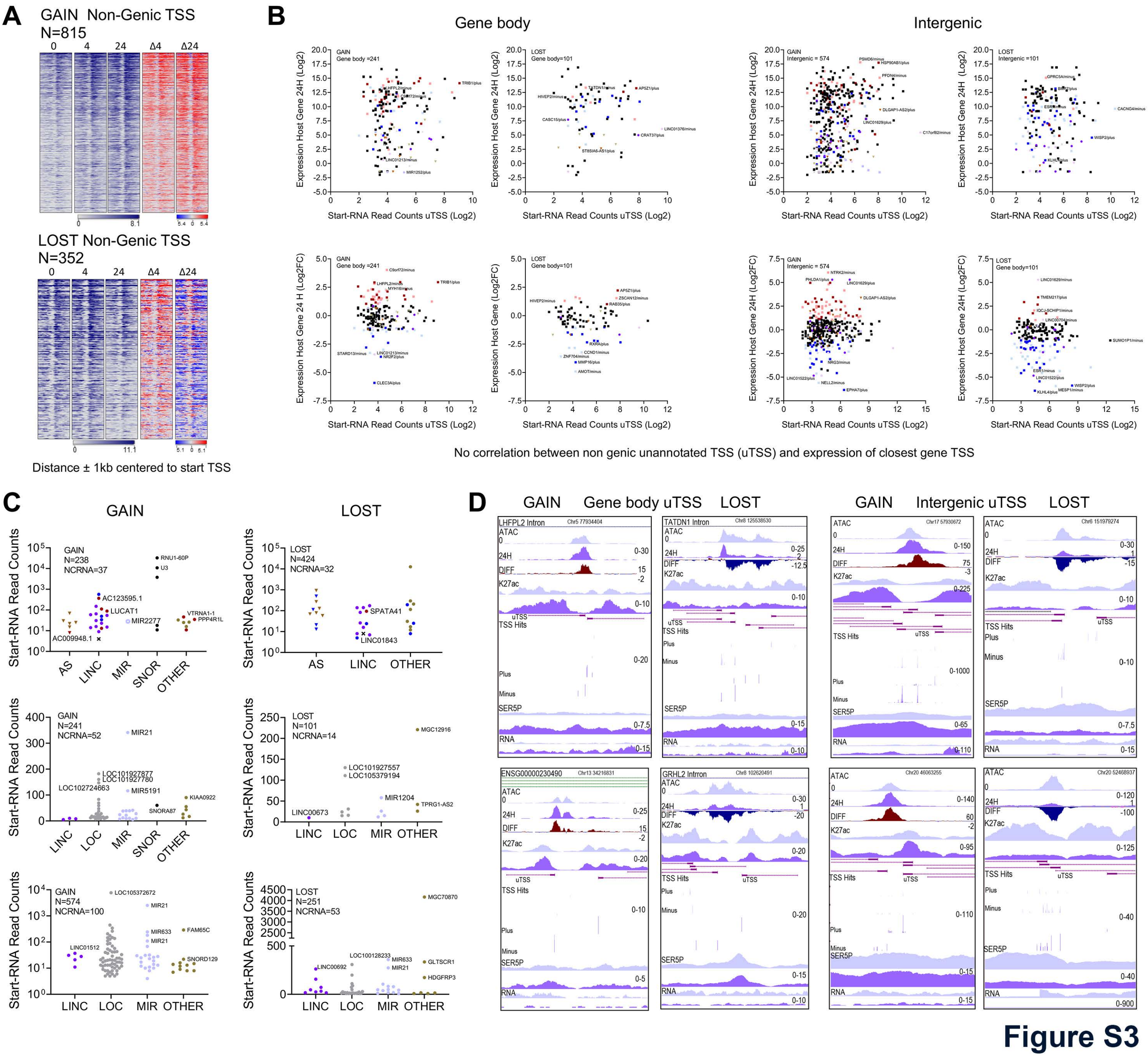
A
GAIN Non-Genic TSS
N=815
LOST Non-Genic TSS
N=352
Distance ± 1kb centered to start TSS
B
Gene body
Intergenic
Expression Host Gene 24H (Log2)
Start-RNA Read Counts uTSS (Log2)
Expression Host Gene 24 H (Log2FC)
No correlation between non genic unannotated TSS (uTSS) and expression of closest gene TSS
C
GAIN
LOST
Start-RNA Read Counts
AS
LINC
MIR
SNOR
OTHER
LOC
D
GAIN
Gene body uTSS
LOST
Intergenic uTSS
ATAC
DIFF
K27ac
TSS Hits
Plus
Minus
SER5P
RNA
Figure S3

Figure S3. Proteasome inhibition drives non-genic transcription that is largely non-coding.
A) Heatmap showing H3K27ac signal at nongenic TSS. H3K27ac signal is sorted descending order based on total start-TSS read signal. N is the number of nongenic TSS within each DOCR. **B)** Scatter plot showing correlation between expression of nongenic TSS (start-seq) and closest gene (RNA-seq) read counts. **C)** Graph showing expression of different classes of noncoding RNAs nearby genic and nongenic TSS. **D)** Browser tracks showing examples of nongenic TSS in the gene body and intergenic GAIN and LOST DOCRs. Tracks show read coverage of chromatin accessibility (ATAC), Differential ATAC (DIFF), H3K27ac, start RNA hits, strand, Ser5P and RNA expression. Chromosome start coordinate for each TSS is shown. Gene names closest to the nongenic TSS in gene body are indicated. Tracks: Control (0), MG24H (24H).
