# Supplementary material for: Proteasome Inhibition Reprograms Chromatin Landscape in Breast Cancer
Source: Cancer Res Commun. 2024 Apr 16;4(4):1082–99. doi: 10.1158/2767-9764.CRC-23-0476 (PMC11019832; doi:10.1158/2767-9764.CRC-23-0476)
Supplement: Figure S4 — Shows biological processes affected by MG132 treatment. [file crc-23-0476-s04.pdf]

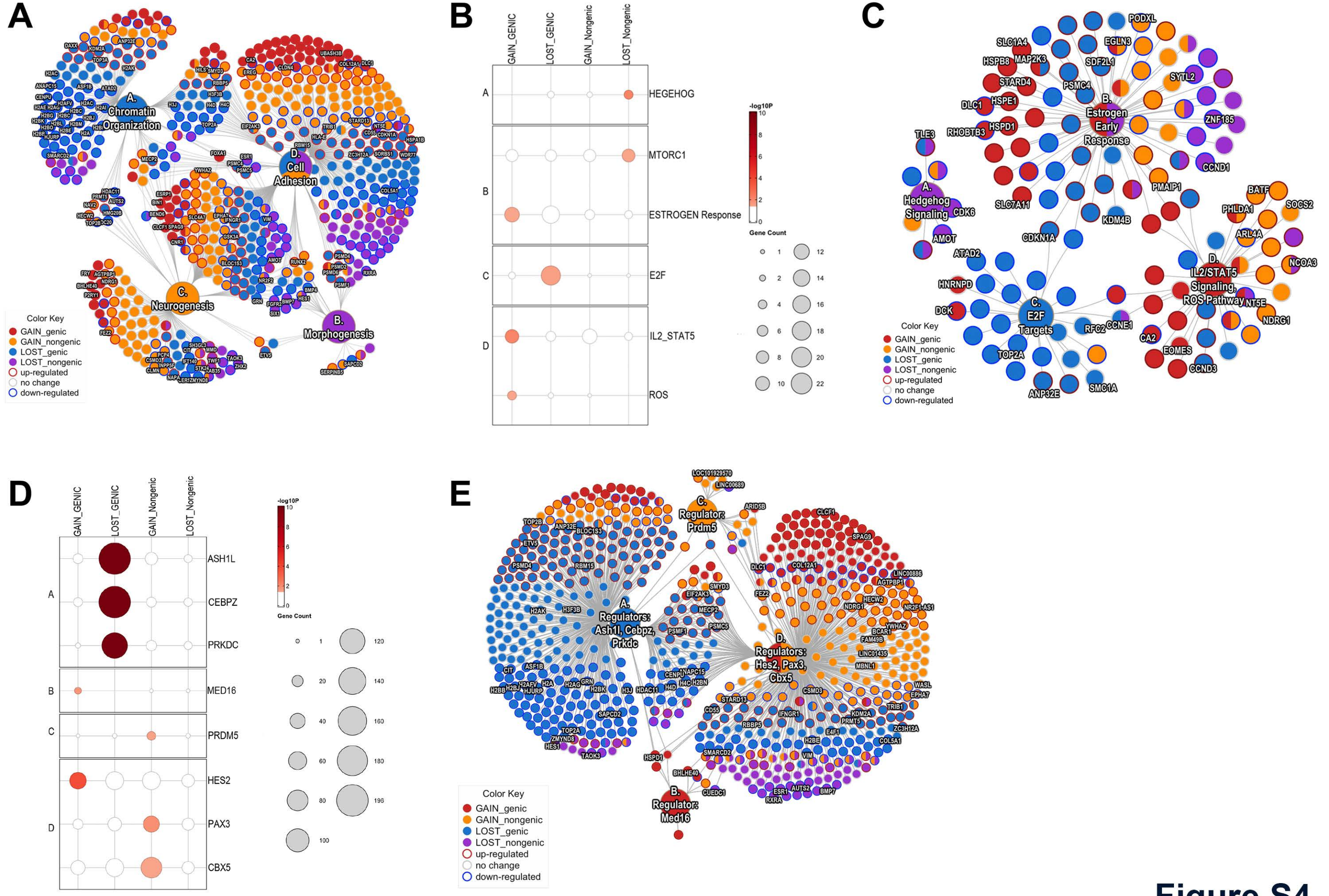
A
A. Chromatin Organization
B. Morphogenesis
C. Neurogenesis
D. Cell Adhesion
Color Key
GAIN_genic
GAIN_nongenic
LOST_genic
LOST_nongenic
up-regulated
no change
down-regulated
B
GAIN_GENIC
LOST_GENIC
GAIN_Nongenic
LOST_Nongenic
HEGEHOG
MTORC1
ESTROGEN Response
E2F
IL2_STAT5
ROS
-log10P
Gene Count
C
A. Hedgehog Signaling
B. Estrogen Early Response
C. E2F Targets
D. IL2/STAT5 Signaling, ROS Pathway
Color Key
GAIN_genic
GAIN_nongenic
LOST_genic
LOST_nongenic
up-regulated
no change
down-regulated
D
GAIN_GENIC
LOST_GENIC
GAIN_Nongenic
LOST_Nongenic
ASH1L
CEBPZ
PRKDC
MED16
PRDM5
HES2
PAX3
CBX5
-log10P
Gene Count
E
A. Regulators: Ash1l, Cebpz, Prkdc
B. Regulator: Med16
C. Regulator: Prdm5
D. Regulators: Hes2, Pax3, Cbx5
Color Key
GAIN_genic
GAIN_nongenic
LOST_genic
LOST_nongenic
up-regulated
no change
down-regulated

Figure S4

Figure S4. Biological processes affected by MG132 treatment

A) cNet-plot showing GO biological processes that were significantly enriched for genes closest to genic and nongenic TSS. The color of gene node represents the most enriched DOCR as per legend, genes in each node are organized based on direction of expression, border color of the dot identifies genes UP (red) or DOWN (blue) ($log2FC \leq 1$, FDR=0.05) in cells treated for 24H. **B)** Enriched hallmark pathways. Dot plot showing the degree of enrichment for genes closest to DOCRs split by category. Dot size represents the number of genes in each hallmark pathway. **C)** cNet-plot showing hallmark pathways enriched for genes closest to genic and non-genic TSS. Key genes in each pathway are shown. **D)** Transcriptional cofactors predicted to regulate genes closest to genic and nongenic TSS. Dot plot showing the degree of enrichment for genes closest to DOCRs split by category. Dot size represents the number of genes predicted to be regulated by each cofactor as determined from GTRD (gene transcription regulation database. **E)** cNet-plot showing GTRD factors whose targets are enriched for genes closest to genic and non-genic TSS.
