# Supplementary material for: Proteasome Inhibition Reprograms Chromatin Landscape in Breast Cancer
Source: Cancer Res Commun. 2024 Apr 16;4(4):1082–99. doi: 10.1158/2767-9764.CRC-23-0476 (PMC11019832; doi:10.1158/2767-9764.CRC-23-0476)
Supplement: Figure S5 — Shows Effects of MG132 on accessibility of breast cancer specific super enhancers and predicted associated genes. [file crc-23-0476-s05.pdf]

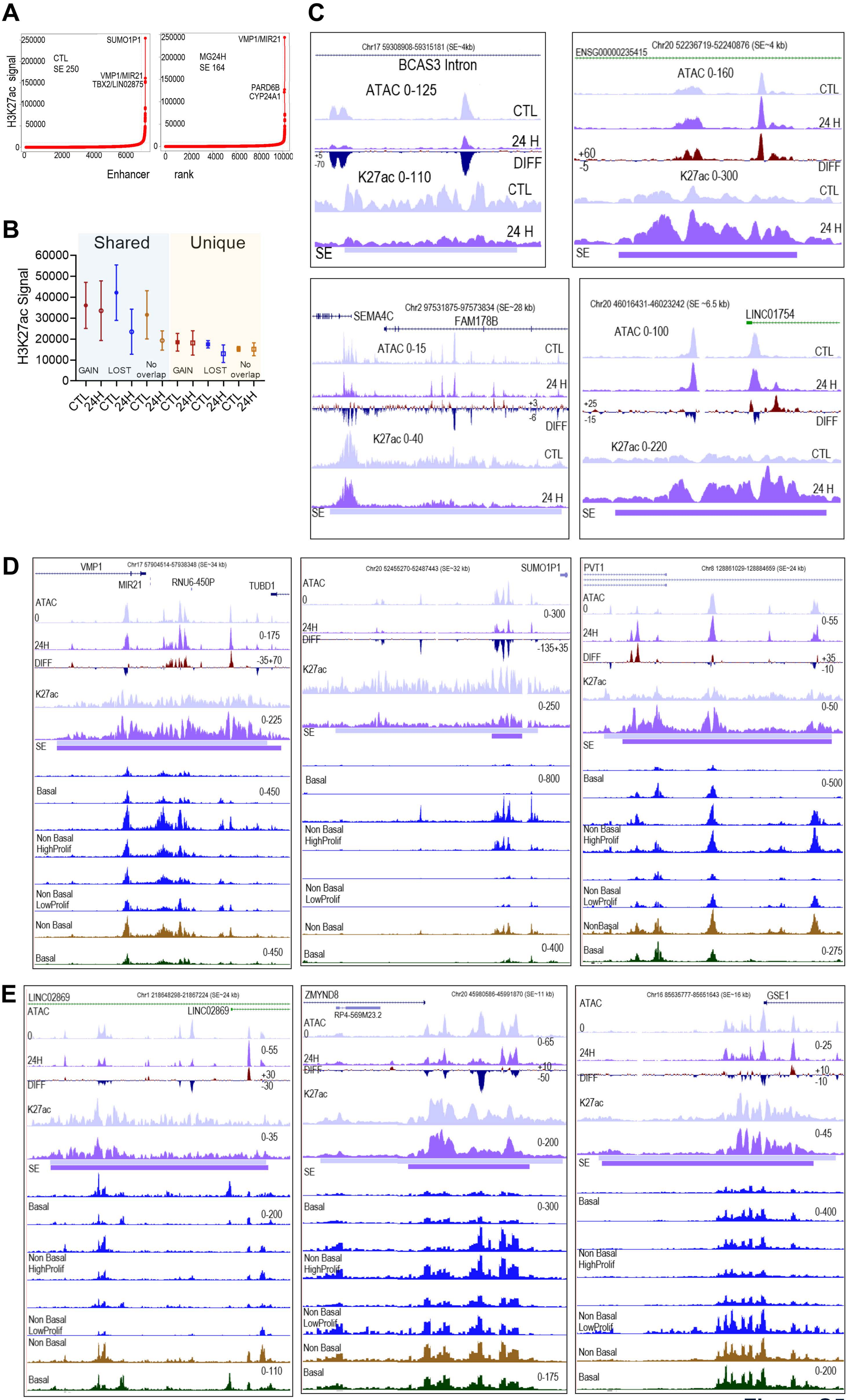
A
H3K27ac signal
CTL
SE 250
SUMO1P1
VMP1/MIR21
TBX2/LINC02875
MG24H
SE 164
VMP1/MIR21
PARD6B
CYP24A1
Enhancer rank
B
Shared
Unique
H3K27ac Signal
GAIN
LOST
No overlap
CTL
24H
C
Chr17 59308908-59315181 (SE~4kb)
BCAS3 Intron
ATAC 0-125
K27ac 0-110
DIFF
SE
Chr20 52236719-52240876 (SE~4 kb)
ENSG00000235415
ATAC 0-160
K27ac 0-300
Chr2 97531875-97573834 (SE~28 kb)
SEMA4C
FAM178B
ATAC 0-15
K27ac 0-40
Chr20 46016431-46023242 (SE ~6.5 kb)
LINC01754
ATAC 0-100
K27ac 0-220
D
VMP1
Chr17 57904514-57938348 (SE~34 kb)
MIR21
RNU6-450P
TUBD1
Chr20 52455270-52487443 (SE~32 kb)
SUMO1P1
PVT1
Chr8 128861029-128884659 (SE~24 kb)
ATAC
K27ac
Basal
Non Basal HighProlif
Non Basal LowProlif
Non Basal
E
LINC02869
Chr1 218648298-21867224 (SE~24 kb)
ZMYND8
Chr20 45980586-45991870 (SE~11 kb)
RP4-569M23.2
Chr16 85635777-85651643 (SE~16 kb)
GSE1

Figure S5

Figure S5 . Proteasome inhibition affects accessibility of super enhancers important in breast cancer biology.

A) Identification of super enhancers from control and MG132 treated MCF-7 cells. Enhancer regions are plotted in an increasing order based on normalized H3K27ac signal. Super-enhancers are defined as the population of enhancers above the inflection point of the curve. Examples of closest TSS to 3 top SEs in each class are shown **B)** Graph showing the level of H3K27ac signal observed at shared and unique super enhancers that overlap or do not overlap with DOCRs. **C)** Browser tracks showing examples of SEs 1) BCAS3 intron unique to control and overlaps with LOST DOCR; 2) Chr20 desert unique to MG24H, overlaps with GAIN; 3) SEMA4C/FAM178B unique to control and LINCO1754 unique to MG24H do not overlap with DOCRs. Tracks show read coverage of chromatin accessibility (ATAC), differential ATAC (DIFF), H3K27ac, and SE region. SE chromosome start coordinates are shown on top. Tracks: Control (0), MG24H (24H). **D)** SE show individual tumor heterogeneity in DNA accessibility. Browser tracks of VMP1/MIR21 (Chr 17), SUMO1P1 (Chr 20) and PVT1 (Chr 8), SE regions are representative examples, cluster A, B and D. Tracks show read coverage of chromatin accessibility (ATAC), differential ATAC (DIFF), H3K27ac, SE, normalized ATAC signal for individual tumors showing High or Low proliferation, average normalized ATAC signal for non-basal (brown) and basal (green) breast tumors. Tracks: Control (0), MG24H (24H). **E)** Browser tracks of C1orf143 (LINC02869, Chr1), ZMYND8 (Chr 20), GSE1 (Chr 16), SE regions are representative examples, cluster C, E and F.
