# Supplementary material for: Proteasome Inhibition Reprograms Chromatin Landscape in Breast Cancer
Source: Cancer Res Commun. 2024 Apr 16;4(4):1082–99. doi: 10.1158/2767-9764.CRC-23-0476 (PMC11019832; doi:10.1158/2767-9764.CRC-23-0476)
Supplement: Figure S6 — Shows effects of MG132 on estrogen receptor-mediated transcription. [file crc-23-0476-s06.pdf]

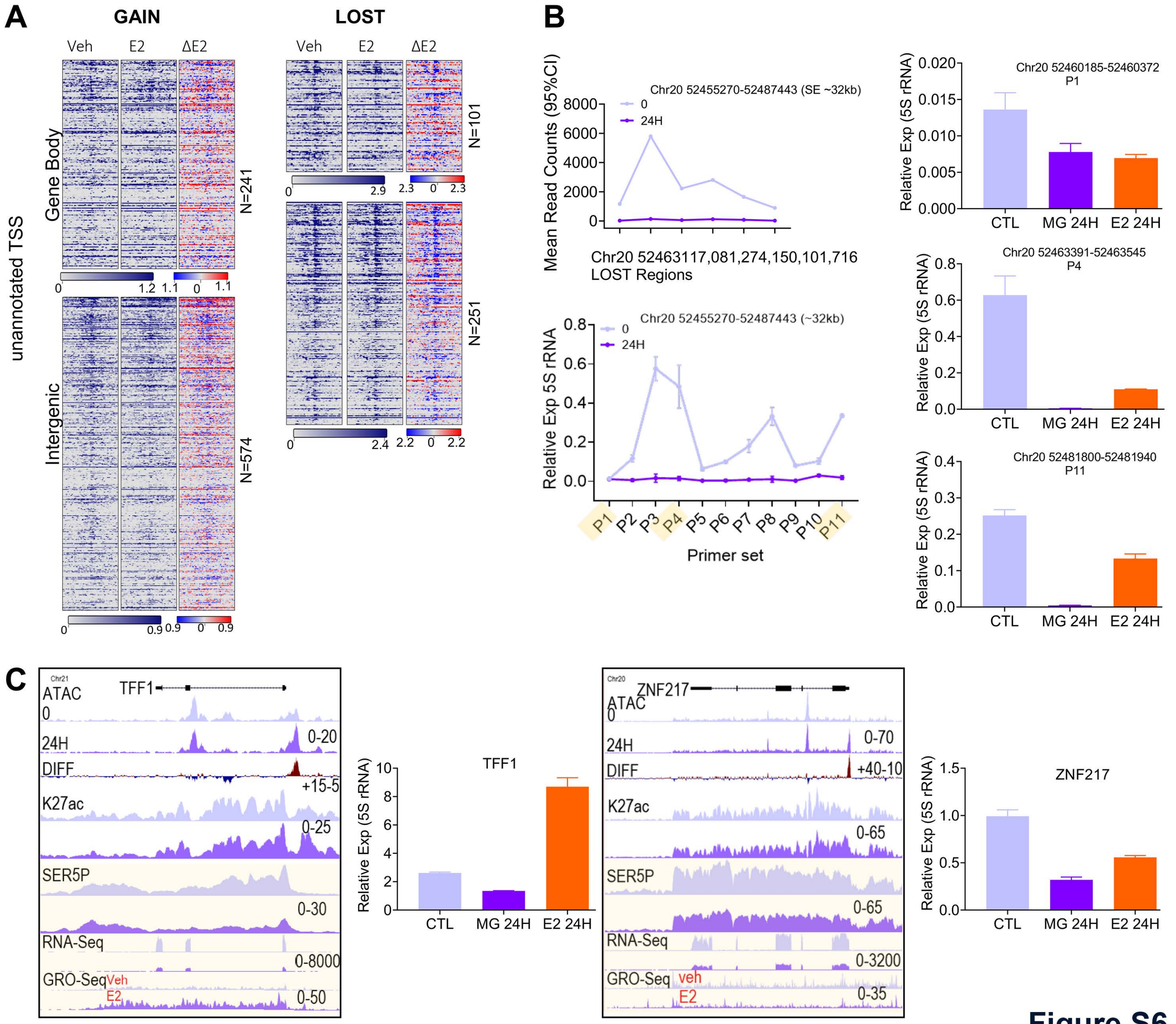
A
GAIN
LOST
Veh
E2
ΔE2
unannotated TSS
Gene Body
Intergenic
N=241
N=574
N=101
N=251
B
Mean Read Counts (95%CI)
Chr20 52455270-52487443 (SE ~32kb)
0
24H
Chr20 52463117,081,274,150,101,716
LOST Regions
Relative Exp 5S rRNA
Chr20 52455270-52487443 (~32kb)
Primer set
P1 P2 P3 P4 P5 P6 P7 P8 P9 P10 P11
Relative Exp (5S rRNA)
Chr20 52460185-52460372
P1
Chr20 52463391-52463545
P4
Chr20 52481800-52481940
P11
CTL
MG 24H
E2 24H
C
Chr21
TFF1
ATAC
0
24H
0-20
DIFF
+15-5
K27ac
0-25
SER5P
0-30
RNA-Seq
0-8000
GRO-Seq
Veh
E2
0-50
TFF1
Chr20
ZNF217
0-70
+40-10
0-65
0-3200
veh
0-35
ZNF217

Figure S6

Figure S6 . Proteasome inhibition affects accessibility of transcriptionally active ER regions.

A) Non-genic DOCR transcription is responsive to E2 treatment. Heatmaps showing GRO-seq signal at nongenic TSSs that overlap GAIN (left) and LOST DOCRs (right) in cells treated with vehicle (veh) and estradiol (E2). **B)** MG132 and E2 repress transcription of SUMO1P1 SE region. Graph showing RNA-seq expression (read counts) of the DOCR regions that overlap with the SUMO1P1 SE. **Bottom**: Graph showing RNA expression from the SE region, x-axis shows the primer sets used qPCR analysis. Right: Graph showing RNA expression of selected regions within SE in cells treated with MG132 and E2. Primer genomic coordinates (P) are indicated **C)** Tracks showing chromatin state and transcription features of TFF1 and ZNF217, representative genes induced and repressed by E2. Graphs show RNA expression TFF1 and ZNF217. RNA expression normalized to 5S rRNA.
