# Supplementary material for: Proteasome Inhibition Reprograms Chromatin Landscape in Breast Cancer
Source: Cancer Res Commun. 2024 Apr 16;4(4):1082–99. doi: 10.1158/2767-9764.CRC-23-0476 (PMC11019832; doi:10.1158/2767-9764.CRC-23-0476)
Supplement: Table S1 — List of resources and experimental reagents used in the study. [file crc-23-0476-s07.docx]

**Table S1: KEY RESOURCES TABLE**

| **REAGENT or RESOURCE** | **SOURCE** | **IDENTIFIER** |
| --- | --- | --- |
| **Antibodies** | | |
| Pol II (8WG16) mouse monoclonal | Santa Cruz Biotechnology | Sc-56767; RRID:AB_785522 |
| Anti-RNA polymerase II CTD repeat YSPTSPS (phospho S5) | Abcam | Ab5131; RRID:AB_449369 |
| Anti-RNA polymerase II CTD repeat YSPTSPS (phospho S2) | Abcam | Ab5095; RRID:AB_304749 |
| Anti-Histone H3 antibody | Abcam | Ab1791; RRID:AB_302613 |
| Anti-Histone H3.3 Antibody | Millipore | 09-838; RRID:AB_10845793 |
| Anti-Histone H3 (tri methyl K4) | Active Motif | 39159; RRID:AB_2615077 |
| Anti-Histone H3 (tri methyl K4) | Abcam | Ab8580; RRID:AB_306649 |
| Anti-Histone H3 (acetyl K27) | Abcam | Ab4729; RRID:AB_2118291 |
| Anti-Histone H3 (acetyl K122) | Abcam | Ab33309; RRID:AB_942262 |
| Anti-Histone H3 (Acetyl-K9/14) | Cell Signaling Technology | 9677; RRID:AB_1147653 |
| Anti-Histone H3 (tri methyl K36) | Abcam | Ab9050; RRID:AB_306966 |
| Anti-ERα (F-10) | Santa Cruz Biotechnology | sc-8002; RRID:AB_627558 |
|  |  |  |
| **Chemicals** | | |
| MG132 | Calbiochem | 474790 |
| HEPES pH 8.0 | AppliChem | A69060125 |
| EGTA, 0.5M | bioWorld | 4052008-2 |
| CaCl2, 1M | Sigma | 21115 |
| Sodium Acetate, 3M | Invitrogen | AM9740 |
| Tris HCL, pH 8.0 | Invitrogen | AM98559 |
| MgCl2 | Invitrogen | AM9530G |
| EDTA, 0.5M | Sigma | E7889 |
| NaCl, 5M | Invitrogen | AM9609 |
| Sodium Deoxycholate | Sigma | 30970 |
| Sucrose | MP Biochemicals | 802536 |
| NP-40 | Fluka | 74385 |
| PMSF | Sigma | P7626-5G |
| Tween-20 | Sigma | P2287 |
| Triton X100 | Sigma | X100 |
| Spermine | Sigma | S-2876 |
| Spermidine | Sigma | S-2501 |
| Glycerol | Sigma | G7893 |
| PIPES Buffer 0.5 M pH 6.8 | bioWorld | 41620033-1 |
| SDS 20% Solution | Ambion | AM9820 |
| Lithium Chloride 1M | Growcells | MRGF-1333 |
| Protease Inhibitors | Roche | 11873580001 |
| Phosphate Inhibitor Cocktail 3 | Sigma | P0044 |
| Phosphatase Inhibitor Cocktail 2 | Sigma | P5726 |
| DDT | Fermentas | RO861 |
| 2-Mercaptoethanol | Sigma | M3148 |
| Bovine Serum Albumin | Sigma | A3059 |
| Non Fat Dry Milk | BIORAD | 170-6404 |
| Tris Glycine Novex WedgeWell^TM^ 4-12% | Invitrogen | XPO4120BOX |
| Tris Glycine Novex Wedgewell 8-16% | Invitrogen | XPO8160BOX |
| BIORAD Protein Assay Dye Reagent Concentrate | BIORAD | 5000006 |
| Tris Glycine SDS Sample Buffer 2X | ThermoFisher | LC2676 |
| PVDF membrane | Invitrogen | LC2002 |
|  |  |  |
| **Experimental Models: Cell Lines** |  |  |
| MCF-7 Breast Cancer Cells | ATCC | HTB-22, RRID:CVCL_0031 |
| MycoAlert® Mycoplasma Detection Kit | LONZA | LT07-318 |
| Dulbecco’s Phosphate Buffered Saline | Invitrogen | 21600-051 |
| Fetal Bovine Serum (FBS) | Atlanta Biologicals | S11150 |
| Fetal Bovine Serum – Charcoal/Dextran Treated | Atlanta Biologicals | S11650 |
| Minimum Essential Media | GIBCO/ThermoFisher | 11090-081 |
| Phenol Red Free Minimum Essential Media | GIBCO/ThermoFisher | 51200-038 |
| 0.25% Trypsin, 0.1% EDTA | GIBCO/ThermoFisher | 25200-056 |
| L-Glutamate | GIBCO/ThermoFisher | H0887 |
| Penicillin Streptomycin | Sigma | P0781 |
| HEPES Solution pH7.0-7.6 | Sigma | P0781 |
| DMSO | Sigma | D8418 |
|  |  |  |
| **Extra Reagents, Enzymes, ChIP, RNA, Start-Seq** |  |  |
| RNAse A | QIAGEN | 158922 |
| Micrococcal Nuclease | Worthington | LS004798 |
| Proteinase K | Invitrogen | 25530-015 |
| T4 RNA Ligase 2 | NEB | M0242S |
| T4 RNA Ligase 1 | NEB | B0216S |
| RNA 5´ Polyphosphatase | Lucigen | RP8092H |
| 5’ Terminator Exonuclease | Lucigen | TER51020 |
| RNA 5´ Pyrophosphohydrolase (RppH) | NEB | M0356 |
| APex™ Heat-Labile Alkaline Phosphatase | EPICENTRE | AP49100 |
| RNaseZap | Invitrogen | AM9780 |
| RNASE inhibitor | Invitrogen | AM2696 |
| Trizol Reagent | Invitrogen | 15596026 |
| Urea-TBE gel 15% | Novex | EC6885BOX |
| TBE gels 6% | Novex | EC6265BOX |
| Cellulose Acetate Spin Filters | Agilent | 5185-5990 |
| Oligo Clean and Concentrator Kit | Zymo | D4060 |
| Low Range ssRNA Ladder | NEB | N0364 |
| Gel Loading Buffer II | Invitrogen | AM8546G |
|  |  |  |
| **Critical Commercial Assays** |  |  |
| Total RNA Purification Plus Kit | NORGEN BIOTEK | 48400 |
| RNA 6000 kit | Agilent | 5067-1512 |
| RNeasy Mini Kit | QIAGEN | 74104 |
| MinElute PCR Purification Kit | Qiagen | 28004 |
| QIAquick PCR Purification Kit | Qiagen | 281104 |
| NEXTFlex Rapid DNA-Seq Kit | BIOO Scientific | 5144-02 |
| NEBNext® High-Fidelity 2X PCR Master Mix | NEB | M0541 |
| Nextra DNA Kit | Illumina | 15028212 |
| KAPA HiFi Hotstart uracil+ReadyMix PCR Kit | KAPA Biosystems | KK2801 |
| Quant-iT dsDNA HS Assay Kits | Invitrogen | 32854 |
| SuperScript II First-Strand Synthesis System | Invitrogen | 18064014 |
| TruSeq Small RNA Library Prep Kit | Illumina | RS-200-0012 |
| TD buffer NEXTERA Kit | Illumina | FC-121-1030 |
| TDE1 NEXTERA kit | Illumina | FC-121-1030 |
| DynaBeads^TM^ Protein A | Invitrogen | 10002D |
| DynaBeads^TM^ Protein G | Invitrogen | 10004D |
| AMPureXP SPRI Beads | Beckman Coulter | A63881 |
|  |  |  |
| Oligonucleotides | This study | Upon request |
|  | | |
| **Deposited Data** | | |
| ALL data (ChIP-seq, histone modifications and RNAPII) | This study & ref 24 | GSE241601 |
| ATAC-seq | This study | GSE241597 |
| H3K4me1 | This study | GSE241598 |
| Start-RNA-seq | This study | GSE241599 |
| RNA-seq | This study | GSE241600 |
|  |  |  |
| **Public Data Sets** |  |  |
| ATAC-seq (TCGA BRCA samples) | Ref 39 | https://gdc.cancer.gov/about-data/publications/ATACseq-AWG  https://api.gdc.cancer.gov/data/f1c06cd3-cf35-41cc-bc75-6db273c94273 |
| ER-ChIP-seq (GSE99680) | Ref 41 |  |
| Tumor ER | Ref 41 | GSM2654035-GSM2654044 |
| Tumor Input | Ref 41 | GSM2654059-GSM2654068 |
| Normal ER and Input | Ref 41 | GSM2654075-GSM2654080 |
| MCF7-ER | Ref 41 |  |
| Vehicle | Ref 41 | GSM3595411, 413 |
| E2 (45 Minutes) | Ref 41 | GSM3595410, 412 |
| Input | Ref 41 | GSM3595420 |
|  |  |  |
| **GRO-Seq (**GSE41324) | Ref 48 |  |
| Vehicle | Ref 48 | GSM1014637 GSM1014638 GSM1014639 |
| E2 (40 Minutes) | Ref 48 | GSM1014645 GSM1014646 GSM1014647 |
|  | | |
| **Software and Algorithms** | | |
| R x64 statistical computing platform v3.6.1 | The Comprehensive R Archive Network | https://cran.r-project.org/ |
| Prism | GraphPad Software | https://www.graphpad.com |
| R/Bioconductor packages: Biobase_2.46.0, genefilter_1.44, limma_3.42.2, ComplexHeatmap_2.15.4, circlize_0.4.15, clusterProfiler_3.14.3 | Bioconductor | https://www.bioconductor.org/ |
| Cutadapt | Martin, 2011 | https://cutadapt.readthedocs.io/en/stable/  RRID:SCR_011841 |
| Bowtie | Langmead et al., 2009 | http://bowtie-bio.sourceforge.net/index.shtml  RRID:SCR_005476 |
| Bedtools | Quinlan and Hall, 2010 | <https://bedtools.readthedocs.io/en/latest/>  RRID:SCR_006646 |
| deepTools | Ramírez et al, 2016 | https://deeptools.readthedocs.io/en/develop/index.html |
| STAR | Dobin et al., 2013 | <http://code.google.com/p/rna-star/>  RRID:SCR_004463 |
| featureCounts | Liao et al., 2014 | http://subread.sourceforge.net  RRID:SCR_012919 |
| DESeq2 | Love et al., 2014 | http://www.bioconductor.org/packages/release/bioc/html/DESeq2.html  RRID:SCR_000154 |
| MACS2 | Zhang et al., 2008 | https://pypi.org/project/MACS2/ |
| MEME | Bailey et al., 2015 | https://meme-suite.org/meme/ |
| Tobias | Bentsen et al., 2020 | https://github.molgen.mpg.de/pages/loosolab/www/software/TOBIAS/ |
| ROSE | Whyte et al., 2013 | http://younglab.wi.mit.edu/super_enhancer_code.html |
| TSScall | Scruggs et al., 2015 | https://github.com/lavenderca/TSScall |
